# Identification of lung adenocarcinoma subtypes and predictive signature for prognosis, immune features, and immunotherapy based on immune checkpoint genes

**DOI:** 10.3389/fcell.2023.1060086

**Published:** 2023-05-10

**Authors:** Linbin Hua, Jiyue Wu, Jiashu Ge, Xin Li, Bin You, Wei Wang, Bin Hu

**Affiliations:** ^1^ Department of Thoracic Surgery, Beijing Institute of Respiratory Medicine and Beijing Chaoyang Hospital, Capital Medical University, Beijing, China; ^2^ Department of Urology, Beijing Chaoyang Hospital, Capital Medical University, Beijing, China

**Keywords:** lung adenocarcinoma (LUAD), immune checkpoints, prognosis, immunotherapy, individualized treatment

## Abstract

**Background:** Lung adenocarcinoma (LUAD) is the most common variant of non–small cell lung cancer (NSCLC) across the world. Recently, the rapid development of immunotherapy has brought a new dawn for LUAD patients. Closely related to the tumor immune microenvironment and immune cell functions, more and more new immune checkpoints have been discovered, and various cancer treatment studies targeting these novel immune checkpoints are currently in full swing. However, studies on the phenotype and clinical significance of novel immune checkpoints in LUAD are still limited, and only a minority of patients with LUAD can benefit from immunotherapy.

**Methods:** The LUAD datasets were downloaded from The Cancer Genome Atlas (TCGA) and the Gene Expression Omnibus (GEO) databases, and the immune checkpoints score of each sample were calculated based on the expression of the 82 immune checkpoints-related genes (ICGs). The weighted gene co-expression network analysis (WGCNA) was used to obtain the gene modules closely related to the score and two different LUAD clusters were identified based on these module genes by the Non-negative Matrix Factorization (NMF) Algorithm. The differentially expressed genes between the two clusters were further used to construct a predictive signature for prognosis, immune features, and the response to immunotherapy for LUAD patients through a series of regression analyses.

**Results:** A new immune checkpoints-related signature was finally established according to the expression of 7 genes (FCER2, CD200R1, RHOV, TNNT2, WT1, AHSG, and KRTAP5-8). This signature can stratify patients into high-risk and low-risk groups with different survival outcomes and sensitivity to immunotherapy, and the signature has been well validated in different clinical subgroups and validation cohorts.

**Conclusion:** We constructed a novel immune checkpoints-related LUAD risk assessment system, which has a good predictive ability and significance for guiding immunotherapy. We believe that these findings will not only aid in the clinical management of LUAD patients but also provide some insights into screening appropriate patients for immunotherapy.

## Introduction

Lung cancer is one of the most common cancers and the leading cause of cancer death worldwide. Lung cancer has been the subject of several studies to improve its management, including more accurate diagnosis and improved treatment (Thai et al, 2021). Non-small cell lung cancer (NSCLC) accounts for 83% of lung cancers, and lung adenocarcinoma (LUAD) is the predominant subtype of NSCLC with a consistently high incidence (Kadara et al, 2012; Bray et al, 2018). Surgical lobectomy is the most ideal treatment for patients with early LUAD. However, 10%–44% of these patients have a poor prognosis 5 years after surgery (Neal et al, 2019). In addition, since lung adenocarcinoma is usually found at an advanced stage or even with metastases, only radiotherapy, conventional chemotherapy, or immunotherapy can be used, but drug insensitivity still leads to poor prognosis (Denisenko et al, 2018). Therefore, it is of great significance to construct an effective and reliable prognostic signature for LUAD patients to help the early diagnosis of LUAD and reasonable treatment plans to treat different patients, so that patients can receive more suitable treatment for their conditions and get optimum treatment results accordingly.

Including active immunotherapy, passive immunotherapy, immune checkpoint blockade, etc., tumor immunotherapy is becoming a hot topic in tumor treatment and is continuously being discussed. Numerous studies have reported that the human immune-related system can play a critical role in the development and progression of aggressive cancers (Angell and Galon, 2013; Gentles et al, 2015). LUAD is the most studied subtype of lung cancer, and studies have identified its genomic changes and operable mutations (O’Brien et al, 2018). Therefore, immunotherapy targeting immune-related antigens produced by LUAD may be a potentially effective treatment. In recent years, the rapid development of immune checkpoint inhibitors (ICIs), represented by targeting programmed cell death 1 (PD-1)/programmed cell death ligand 1 (PD-L1) and cytotoxic T lymphocyte antigen 4 (CTLA4), has brought a new dawn for the treatment strategy of LUAD (Reck et al, 2022). It is reported that patients with LUAD who received immunotherapy had a median survival of 13 months longer than those who received chemotherapy (Reck et al, 2021). In addition, the US Food and Drug Administration (FDA) and the European Medicines Agency (EMA) have currently approved various ICIs for the treatment of advanced NSCLC (Reck et al, 2022). However, due to the presence of tumor heterogeneity and the complexity of carcinogenic mechanisms, immunotherapy is only applicable to limited patients, and there are significant individual differences in therapeutic effects (Rooney et al, 2015; Li et al, 2016). Thus, it is of great clinical significance to assess the responsiveness of different LUAD patients to immunotherapy to achieve optimal and personalized treatment to improve patient outcomes.

In our study, we conducted a comprehensive analysis of ICGs in lung adenocarcinoma. Based on 82 ICGs, we used the “GSVA” R package to calculate the ICs score for each lung adenocarcinoma sample, and the WCGNA algorithm was used to select module genes significantly associated with ICs scores. Based on the expression levels of these genes, we used the NMF machine learning method to classify LUAD samples into two subgroups with different prognoses and immune characteristics. Based on the immune-related genes differentially expressed between subgroups, we further used COX and LASSO algorithms to construct a new hierarchical signature containing 7 genes. The nomogram constructed based on this signature can accurately and reliably predict the prognosis of LUAD patients in the TCGA database. Besides, LUAD patients classified based on this signature are greatly different in the sensitivity of immunotherapy and targeted therapy. Overall, the signature is helpful for doctors to make early diagnosis and prognosis judgments for LUAD patients and provides some theoretical basis for individualized treatment.

## Materials and methods

### LUAD datasets and sample extraction

We firstly downloaded RNA sequencing data, gene copy data, somatic mutation data, and accompanying clinical information of LUAD patients from The Cancer Genome Atlas (TCGA) database. The data type of the TCGA database is TPM, and the id conversion method is to convert the ID of the Ensemble annotation library to a molecular name (Symbol). Similarly, we obtained the gene expression matrix and clinical information of LUAD patients in GSE72094 (Schabath et al, 2016) and GSE41271 (Sato et al, 2013) microarray datasets from the Gene Expression Omnibus (GEO) database. The “IMvigor210CoreBiologies” R package (http://research-pub.gene.com/IMvigor210CoreBiologies) was utilized to obtain transcriptome data and clinical information of patients with metastatic urothelial carcinoma who received immunotherapy to verify the efficiency of our signature in predicting the effectiveness of immunotherapy (Bellmunt et al, 2016). A total of 82 ICGs involved in the analysis of this study were summarized from previously published studies.

**FIGURE 1:**
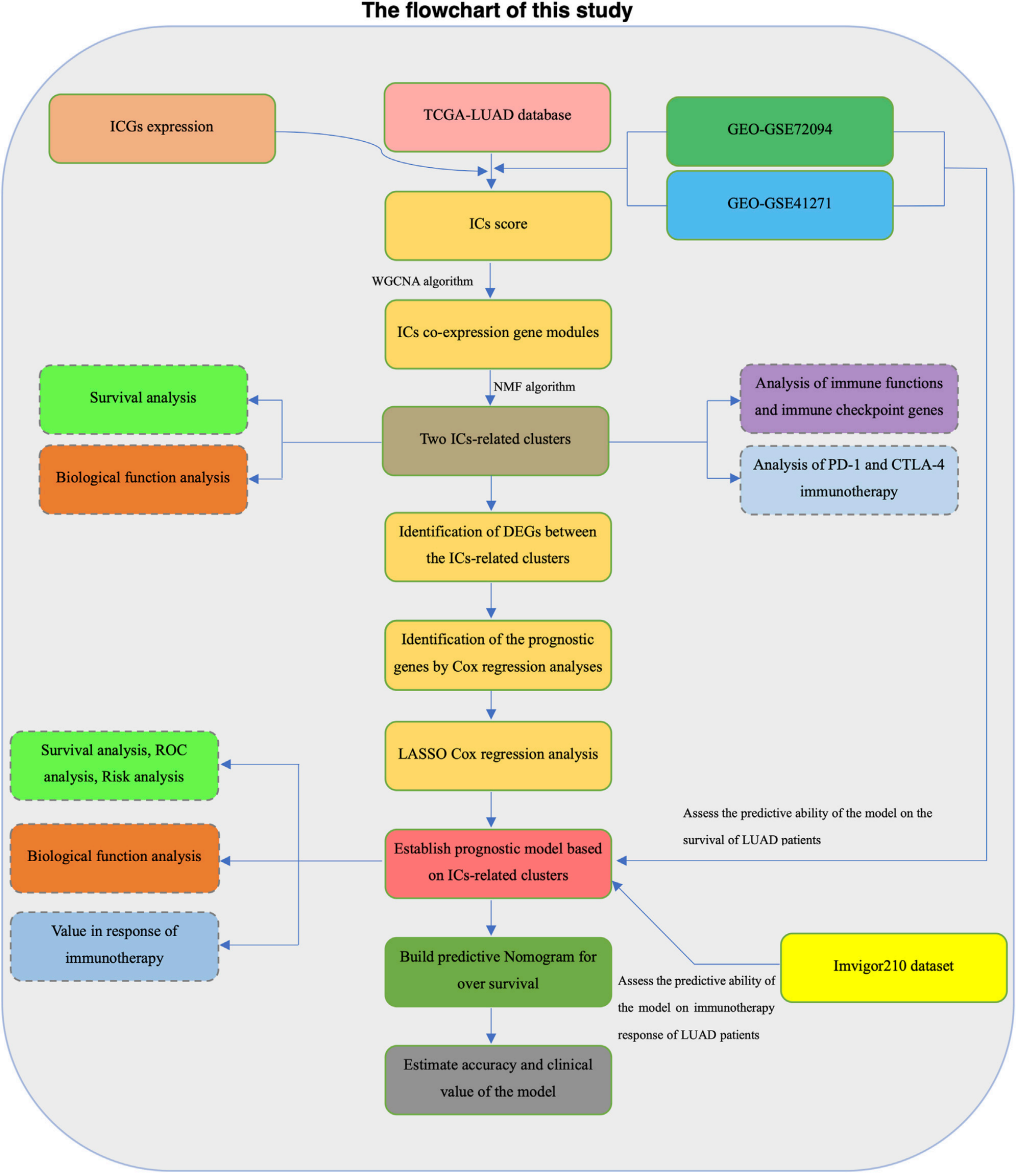
The flowchart of this study.

Samples with incomplete transcriptome data and clinical information in each dataset were excluded from further analysis. We transformed RNA sequencing data in the TCGA-LUAD dataset into log2 (TPM+1) to maintain comparability with the microarray dataset. The screening threshold to filter lowly expressed genes is that the average TPM expression value of all samples is greater than 0.1. The “sva” R package was used to eliminate batch effects on microarray datasets (Leek et al, 2012). The Flowchart of the present study design was shown in Figure 1.

### Construction of immune checkpoints (ICs) scores and co-expression network

ICs scores were calculated for each sample using the “GSVA” R package based on the expression levels of 82 ICGs in the TCGA, GSE72094, and GSE41271 datasets (Hänzelmann et al, 2013). Weighted gene co-expression network analysis (WGCNA) was utilized to identify gene modules with similar expression patterns and analyze the correlation between ICs scores and gene modules (Langfelder and Horvath, 2008). The scale independence and average connectivity of the networks were tested with different power values (from 1 to 20). The appropriate power value was determined when the independent scale was greater than 0.9 and the connectivity was high. Then, the similarity matrix was transformed into a topological matrix with the topological overlap measure (TOM) describing the correlation between genes. The genes were clustered by using 1-TOM as the distance. A dynamic hybrid cutting method was used to establish a hierarchical clustering tree to identify co-expressed gene modules. Each leaf of the tree represents a gene, and genes with similar expression data aggregate to form a branch of the tree and each branch represents a gene module. The correlation between module feature genes and ICs scores was calculated using Pearson correlation analysis, and the genes in the modules most relevant to the ICs scores in the three datasets were intersected.

### Non-negative matrix factorization (NMF) algorithm

Based on the genes obtained by WGCNA analysis, a cluster analysis of the TCGA-LUAD samples was conducted through the “NMF” R package to explore potential molecular subtypes (Devarajan et al, 2015). The “brunet” criterion was selected and iterated 100 times. We set the number of clusters (k) from 2 to 10 and set the minimum number of members of each cluster to 25. We utilized the R package “NMF” to determine the average contour width of the common membership matrix. The cophenetic correlation coefficients (from 0 to 1) were used to reflect the stability of clusters, while the residual sum of squares (RSS) was used to reflect the model’s clustering performance. The optimal k was selected based on the cophenetic, dispersion, and silhouette metrics. LUAD samples were eventually divided into different molecular clusters through the above algorithm and the optimal k.

### Assessment of tumor microenvironment and immune infiltration

The “ESTIMATE” R package was used to estimate the tumor microenvironment, and we obtained the stromal score, immune score, ESTIMATE score, and tumor purity of each sample (Yoshihara et al, 2013). The reference for the signature genes of 23 immune cells were derived from the TISIDB database (http://cis.hku.hk/TISIDB/) (Supplementary Table S1). Based on the signature genes of 23 immune cells, we also utilized the ssGSEA algorithm to assess the infiltration of 23 immune cells in each sample.

### Functional enrichment analysis

Using the “clusterProfiler” R package, we performed Gene ontology (GO) and Kyoto Encyclopedia of Genes and Genomes (KEGG) pathway enrichment analysis (Yu et al, 2012). GSEA analysis was conducted to compare significantly different biological processes between the high-risk group and the low-risk group. Pathways with FDR < 0.25 and p< 0.05 were considered statistically enriched (Subramanian et al, 2005). Using the “GSVA” R package, we performed ssGSEA analysis to evaluate the activity of specific biological pathways in each sample. We used the Spearman correlation test to calculate the correlation between the risk score and the pathway activity, and screen out the pathways with R > 0.3. Reference gene sets included in specific biological pathways were obtained from the MSiDB (https://www.gsea-msigdb.org/gsea/msigdb/index.jsp) database.

### Prediction of the immunotherapy response and drug sensitivity

We used Tumor Immune Dysfunction and Exclusion (TIDE) score to assess the immune escape potential and immunotherapy effect of each LUAD sample (Jiang et al, 2018). The lower the TIDE score, the less likely the tumor is to evade immunity and the more likely the patient will benefit from immunotherapy. To predict the patient’s response to the use of PD-1 and CTLA4 blockers, we also obtained the immunophenotype score (IPS) of LUAD samples from The Cancer Immunome Atlas (TCIA) (Charoentong et al, 2017). Moreover, the correlation between patient risk characteristics and immunotherapy benefits was verified in the IMvigor210 dataset.

The “pRRophetic” R package was used to predict the sensitivity of LUAD patients to targeted drugs. Specifically, with the gene expression profile and IC50 value of cancer cells under the corresponding drug treatment in the GDSC database (https://www.cancerrxgene.org/) as a reference, the IC50 value of targeted drugs is estimated according to the gene expression profile of LUAD samples through the ridge regression of 10 times cross validation.

### Calculation of tumor mutational burden (TMB) and copy number variation (CNV)

TMB represents the number of mutations per million bases in tumor tissue, including base insertions, base deletions, base substitutions, and genetic coding errors. Scholars have proposed that tumor tissues with higher TMB are more easily recognized by the immune system, so immunotherapy against them may be more effective. Therefore, the TMB score of each LUAD patient was calculated based on the somatic mutation data downloaded from the TCGA database. Besides, according to the copy number variation (CNV) data from the TCGA database, we calculated the CNV frequency of the corresponding gene and displayed the results in the form of a lollipop plot.

### Construction of the immune checkpoints-related prognostic stratification signature for LUAD

Differential expression analysis was performed on the two different LUAD clusters obtained by the NMF algorithm, and | log2FC | > 1 and FDR < 0.05 were set as the threshold. The rank sum test was utilized to perform differential expression analysis. The screening threshold for low expression genes is that the average TPM expression value of all samples is greater than 0.1. Repeated samples were averaged using the avereps function of the “limma” R package. Immune-related genes (IRGs) were the result of IRGs union sets downloaded from the Immport (https://www.immport.org/) and InnateDB (https://www.innatedb.ca/) databases. Then we randomly divided the TCGA dataset into the training set and the validation set at a ratio of 7: 3. Using univariate cox regression analysis to identify genes with good prognostic ability in the training set (HR ≠ 1 and *p*-value < 0.05). To further simplified the predictive signature, we used the LASSO regression algorithm in the “glmnet” R package to eliminate overfitting biases through 10-fold cross-validation to obtain a more concise prognostic genes combination (Simon et al, 2011). Finally, we utilized multivariate cox regression to construct the final signature. The coefficient value of each gene is derived, and the risk score is equal to the expression of each gene multiplied by the corresponding regression coefficient.

$$Risk\;Score=\sum_{i=1}^{n}\left({coef}_{i}\times {\mathrm{Exp}}_{i}\right)$$

The time-dependent receiver operating characteristic (ROC) curve was used to assess the predictive power of the above risk signature and the survival differences of patients in different risk groups was compared by the K-M survival curve. Using the same method, the accuracy of the predictive signature was then verified in the validation dataset, the whole dataset, the GSE72094, and the GSE41271 dataset.

### Independent prognostic analysis and nomogram construction

We performed univariate cox and multivariate cox regression analysis on the risk score and some other clinical information and risk scores to screen for independent prognostic factors for LUAD patients. C-index and time-dependent ROC curves were used to evaluate the prognostic efficacy of independent prognostic factors. By using the “regplot” R package, we finally constructed a more accurate predictive nomogram using the independent prognostic factors of LUAD and plotted a calibration curve to assess the prognostic accuracy of the nomogram (Marshall, 2020).

### Cell cultures, RNA extraction and real-time quantitative PCR (RT-qPCR)

Human bronchial epithelial cells (16HBE cells) and human lung cancer cell lines (SPC-A-1 cells, NCI-H1975 cells) were cultured in DMEM (HyClone, United States). All mediums were supplemented with 10% fetal bovine serum (Gibco, United States), 100 U/mL of penicillin and 100 U/mL of streptomycin (Gibco, United States). The conditions of cell cultures were 37°C and 5% CO_2_.

Total RNA were extracted from cultured cells by TRIzol (Invitrogen, Shanghai, China). Reverse transcription reactions were then performed by using a first-strand cDNA synthesis kit (novoprotein, Shanghai, China). Real-time PCR system was configured according to an ABI SYBR Green Master Mix (Applied Biosystems, United States), and the mRNA expressions of genes were detected by using a real-time fluorescent quantitative PCR instrument (QuantStudio 3, Thermo Fisher Scientific, United States). 2^−ΔΔCT^ method was used to caculated the relative expression levels of the genes. GAPDH was used as an internal reference. Primers used in RT-qPCR were listed in Supplementary Table S2.

### RNA interference and RT-qPCR

The SPC-A-1 cells were seeded into plates at an appropriate density. According to the manufacturer’s protocols, small interfering RNA (siRNA, 50–100 nmol/L) and lipofectamine RNAiMAX transfection reagent (Invitrogen, Carlsbad, CA, United States) were used for transfection. Subsequently, RT-qPCR was performed to detect the efficiency of gene knockdown.

### Scratch assay and transwell assay

After the SPC-A-1 cells were transfected for 48 h, 100 mL of Eppen-dorf Tip was used to scratch the cell plate and the cells were washed 2-3 times to remove cellular debris. Observe the changes of cells in each group at 0 h and 48 h with an inverted microscope.

For Transwell migration or invasion assays, 1.5 × 10^4^ cells suspended in medium without serum were seeded in the upper chamber membranes coated without/with Matrigel (BD Biosciences). Then, 600 μL of medium with 10% fetal bovine serum (FBS) was added to the lower chamber. After 24 h, the underside of the membrane was fixed for 30 min and stained with 0.1% crystal violet. The inner side of the membrane was wiped with a cotton swab. Then, the cells were quantified under a microscope.

### Cell viability and cell colony formation assay

The SPC-A-1 cells were divided into groups of Control, SPC-A-1+si RHOV NC, SPC-A-1+si RHOV. After 48 h of transfection, the cells were digested with trypsin and were seeded in a 96-well plate at 4 × 10^4^ cells/well. After 0, 24, and 48 h, 10 μL of Cell Counting Kit-8 (CCK8) (DoJinDo, Japan) was added to each well and incubated at 37°C for 4 h. Then using a microplate reader (Thermo, United States) to measure the absorbance at 450 nm.

The SPC-A-1 cells at the logarithmic growth stage were taken for the preparation of cell suspension and inoculated at 800 cells/well in the six-well plates. According to the experimental grouping, each group had three multiple wells and was cultured for 3 weeks. When visible colony mass appeared in the Petri dish, cell culture was terminated. The supernatant was discarded, 4% paraformaldehyde (Leagene Biotech, DF0135, China) was applied for fixation for 20 min, followed by crystal violet staining (Leagene Biotech, DZ0053, China) for 15 min. The colony formation rate is calculated by (Number of clones/Number of inoculated cells) × 100%.

### Statistical analysis

All statistical analyses in our study were performed by R software (version 4.2.1). The student’s *t*-test was used to compare continuous variables, and the Wilcoxon rank sum test was used to compare non-normal distribution variables. Two groups of categorical variables were compared using the chi-square test. All statistical tests were two-sided, and *p*-value < 0.05 was considered statistically significant.

## Results

### Identification and enrichment analysis of ICs score-related gene modules in LUAD

**FIGURE 2:**
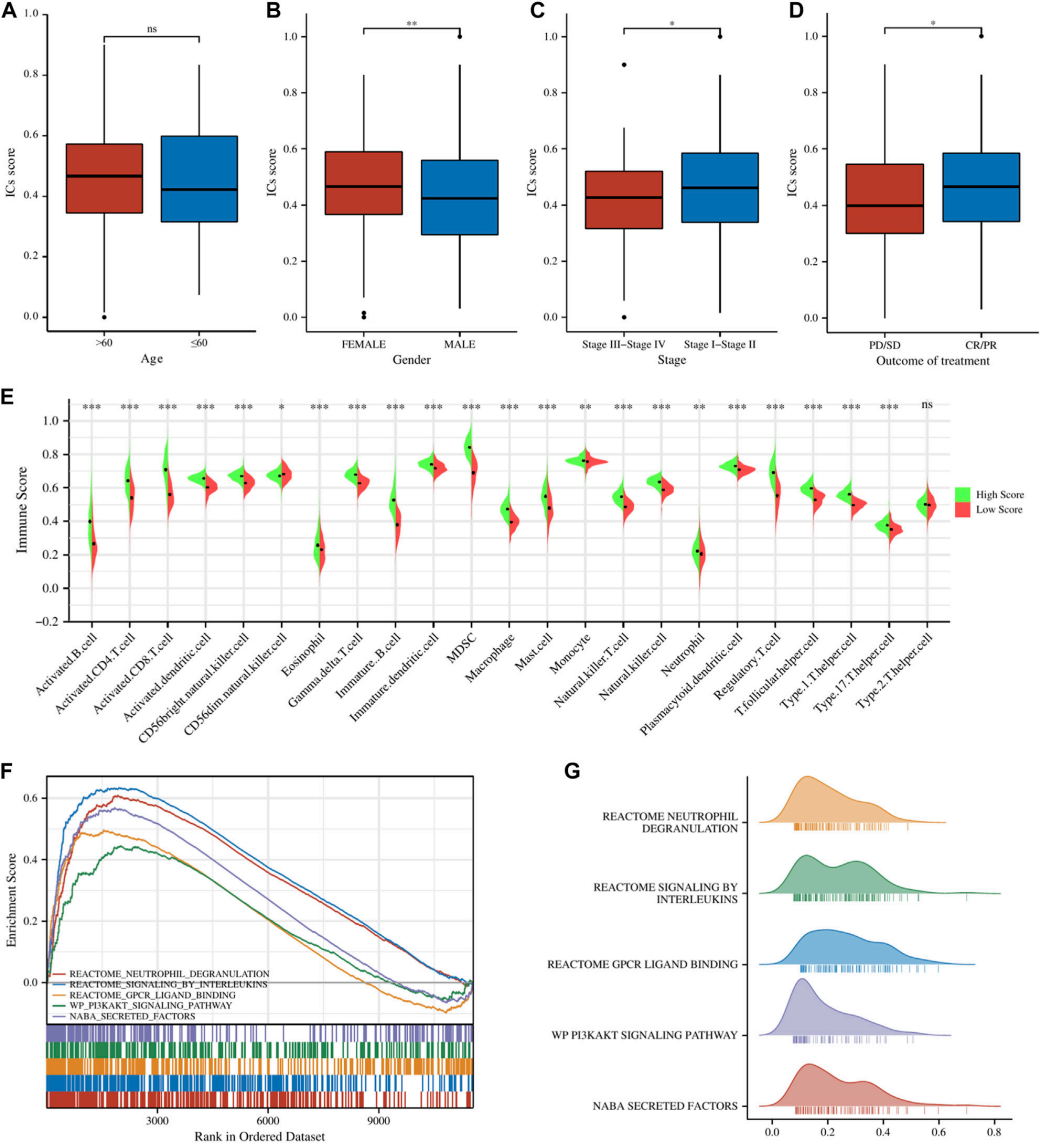
The comparisons of age **(A)**, gender **(B)**, clinical stage **(C)**, and main outcome **(D)** between different ICs score subgroups. **(E)** The pod plot displaying the difference in immune cell infiltration between different ICs score subgroups. **(F, G)** GSEA enrichment analysis between the two subgroups. ns, not significant; *, p < 0.05 ; **, p < 0.01 ; ***, p < 0.001 .

All of the ICGs used for ssGSEA analysis were shown in Supplementary Table S3 and the box plot showed the differential expression of ICGs between different ICs score subgroups in Supplementary Figure S1. The Baseline characteristics of the LUAD patients in this study were shown in Supplementary Table S4. Figures 2A–D showed the relationship between ICs scores and clinical characteristics (including age, gender, clinical stage, and outcome of treatment) of patients in the TCGA-LUAD cohort. It is observed that female patients had significantly higher ICs scores than male patients. Moreover, patients with earlier clinical stages had higher ICs scores, and CR/PR (Complete Response/Partial Response) patients had higher ICs scores than PD/SD (Progressive Disease/Stable Disease) patients. This seems to indicate that ICs score was negatively associated with the progression of lung cancer. Immune cell infiltration results showed that the abundance of various immune cells including B cells, CD4^+^ T cells, CD8^+^ T cells, T regulatory cells, macrophages, etc. was significantly higher in the high ICs score group (Figure 2E), which indicates that immune cell infiltration in the tumor microenvironment was more abundant in patients with high ICs score. Figures 2F–G showed the main results of GSEA enrichment analysis between the above two subgroups. The waterfall graphs further showed the relationship between ICs scores and the mutation status of common driving genes (Supplementary Figure S2).

**FIGURE 3:**
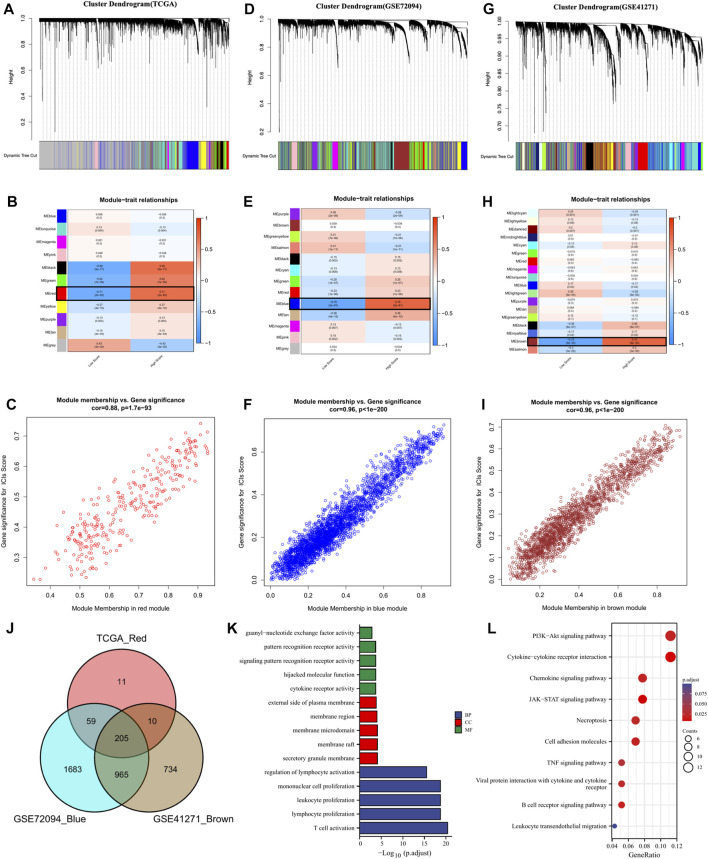
The coexpression network was established using weighted gene coexpression network analysis methods based on the LUAD RNA-seq profiles from **(A)** the TCGA-LUAD database, **(D)** GSE72094 dataset, and **(G)** GSE41271 dataset. Heatmap demonstrating the correlation between module eigengenes and immune checkpoints in the **(B)** TCGA-LUAD dataset, **(E)** GSE72094 dataset, and **(H)** GSE41271 dataset. **(C)** The red module had the strongest correlation with ICs score in the TCGA-LUAD dataset (Cor = 0.88, P=1. 7e−93). **(F)** The blue module had the strongest correlation with ICs score in the GSE72094 cohort (Cor = 0.96, P < 1e−200). **(I)** The brown module had the strongest correlation with ICs score in the GSE41271 dataset (Cor = 0.96, P < 1e−200). **(J)** Venn diagram displaying the ICs score-related selected intersection genes from different datasets. **(K, L)** Gene ontology (GO) and Kyoto Encyclopedia of Genes and Genomes (KEGG) analyses of ICs score-related intersecting genes.

To further screen the genes significantly associated with ICs score, we performed WCGNA analysis. In the TCGA cohort (Figures 3A–C), the red module was most closely related to ICs scores in 11 modules (*R* ^2^ = 0.71, p = 2e-83). In the GSE72094 cohort (Figures 3D–F), the blue module in the 13 modules was most closely related to the ICs score (*R* ^2^ = 0.75, p = 3e-81). In the GSE41271 queue (Figures 3G–I **)**, the brown module in the 18 modules was most closely related to the ICs score (*R* ^2^ = 0.75, p = 9e-35). By intersecting the above three hub gene modules, we totally identified 205 genes significantly related to the ICs score (Figure 3J). The genes belonging to each module of TCGA cohort, GSE72094 cohort and GSE41271 cohort and the results of the intersection of the three modules were shown in Supplementary Table S5. GO enrichment analysis showed that these ICs-associated genes were mainly enriched in immune-related biological processes, such as T cell activation, lymphocyte proliferation, leukocyte proliferation, etc. (Figure 3K). The results of the KEGG pathway enrichment analysis were also enriched in immune-related pathways such as PI3K-Akt signaling pathway, Cytokine-cytokine receptor interaction, Chemokine signaling pathway, JAK-STAT signaling pathway, and TNF signaling pathway (Figure 3L).

### Stratification of LUAD patients based on ICs score-related hub genes

**FIGURE 4:**
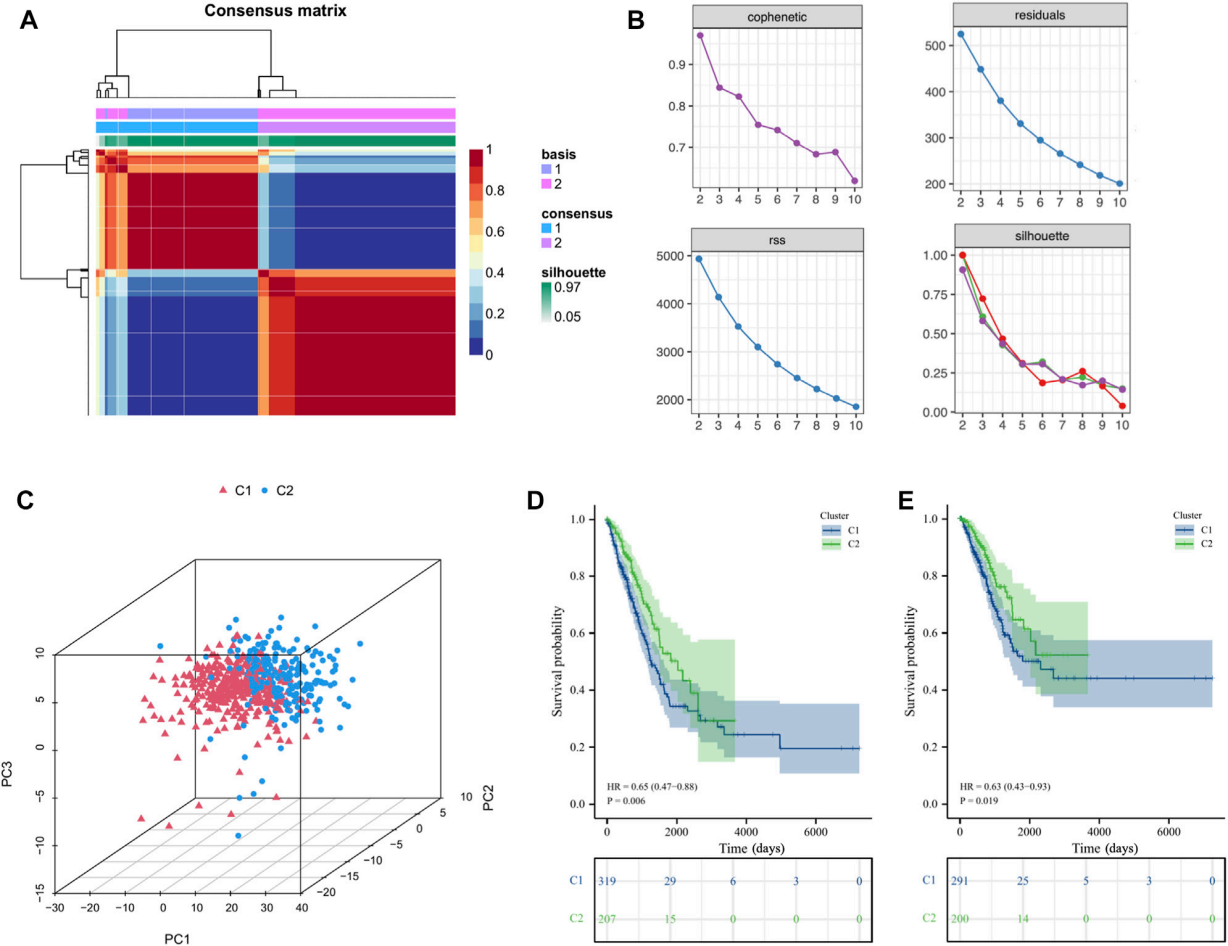
Cluster analysis of intersection genes related to ICs in the TCGA cohort. **(A, B)** According to the steepness of the ' cophenetic ' decline, the LUAD dataset in the TCGA cohort was divided into two distinct clusters when *k* = 2. **(C)** The 3D PCA plots showed the cluster could distinguish lung adenocarcinoma (LUAD) patients based on the expression profiles of the LUAD dataset. **(D, E)** The Kaplan–Meier curve survival analysis of Overall Survival (OS) and Disease-Specific Survival (DSS) between different cluster groups.

Based on the above ICs score-related hub genes, we utilized the NMF algorithm to cluster TCGA-LUAD patients. According to the steepness of the “cophenetic” decline, the optimal number of clusters selected was two (*k* = 2) (Figures 4A, B). PCA analysis showed that the gene expression profile of the C1 (*n* = 319) cluster classified by ICs-related genes was significantly different from that of the C2 (*n* = 207) cluster (Figure 4C). Prognostic analysis showed that LUAD patients in the C2 cluster had significantly better OS (HR = 0.65, p=0.006 ) and DSS (HR = 0.63, p=0.019 ) than LUAD patients in the C1 cluster (Figures 4D, E). The Baseline characteristics of the LUAD patients in the two clusters (C1 and C2) shown in Supplementary Table S6.

### Biological characteristics of ICs related clusters

**FIGURE 5:**
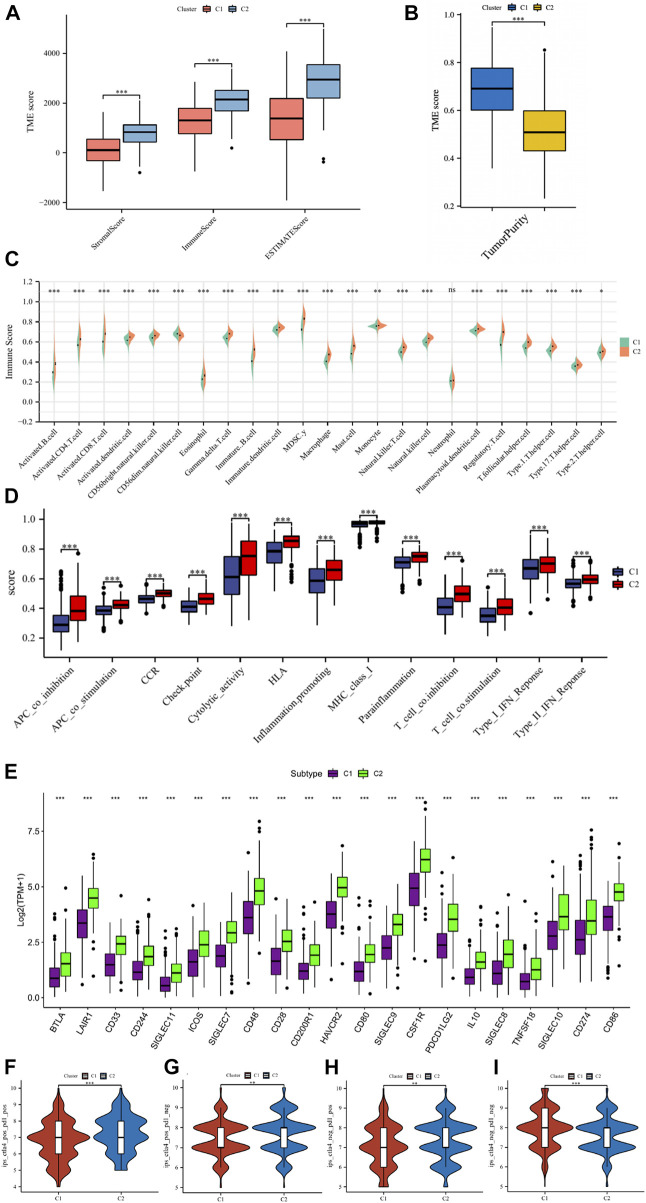
**(A)** The comparisons of the stromal score, immune score, and ESTIMATE score between different clusters. **(B)** The comparisons of tumor purity between different clusters. **(C)** The pod plot displaying the difference in immune cell infiltration between different clusters. **(D)** The boxplot illustrating the difference in immune-related functions between different clusters. **(E)** The boxplot illustrating the difference in the expression of differentially expressed ICs genes between different clusters. The comparison of immunophenotype score (IPS) between different cluster groups. **(F)** CTLA4+_PD1+, **(G)** CTLA4+_PD1−, **(H)** CTLA4−_PD1+, **(I)** CTLA4−_PD1−. ns, not significant; * p < 0.05 ; ** p < 0.01 ; *** p < 0.001 .

The tumor environment and immune cell infiltration of the two clusters were compared to verify the effectiveness of the above clustering method. In terms of the tumor microenvironment, the samples in the C1 cluster had significantly lower stromal scores, immune scores, and ESTIMATE scores and significantly higher tumor purity than the C2 cluster (Figures 5A, B). Correspondingly, the results of immune cell infiltration showed that 23 immune cells except neutrophils were significantly higher in the C2 cluster (Figure 5C). We performed immune function enrichment analysis and found that immune functions were more enriched in the C2 cluster (Figure 5D). As for the expression levels of ICGs, we found that the differentially expressed ICGs were mainly upregulated in the C2 cluster (Figure 5E). IPS analysis can predict the ability of patients to respond to ICIs. We can see that the IPS scores of patients in the C2 cluster were significantly higher than those in the C1 cluster (Figures 5F–I), which means that such patients have stronger immunogenicity and may benefit more from immunotherapy.

**FIGURE 6:**
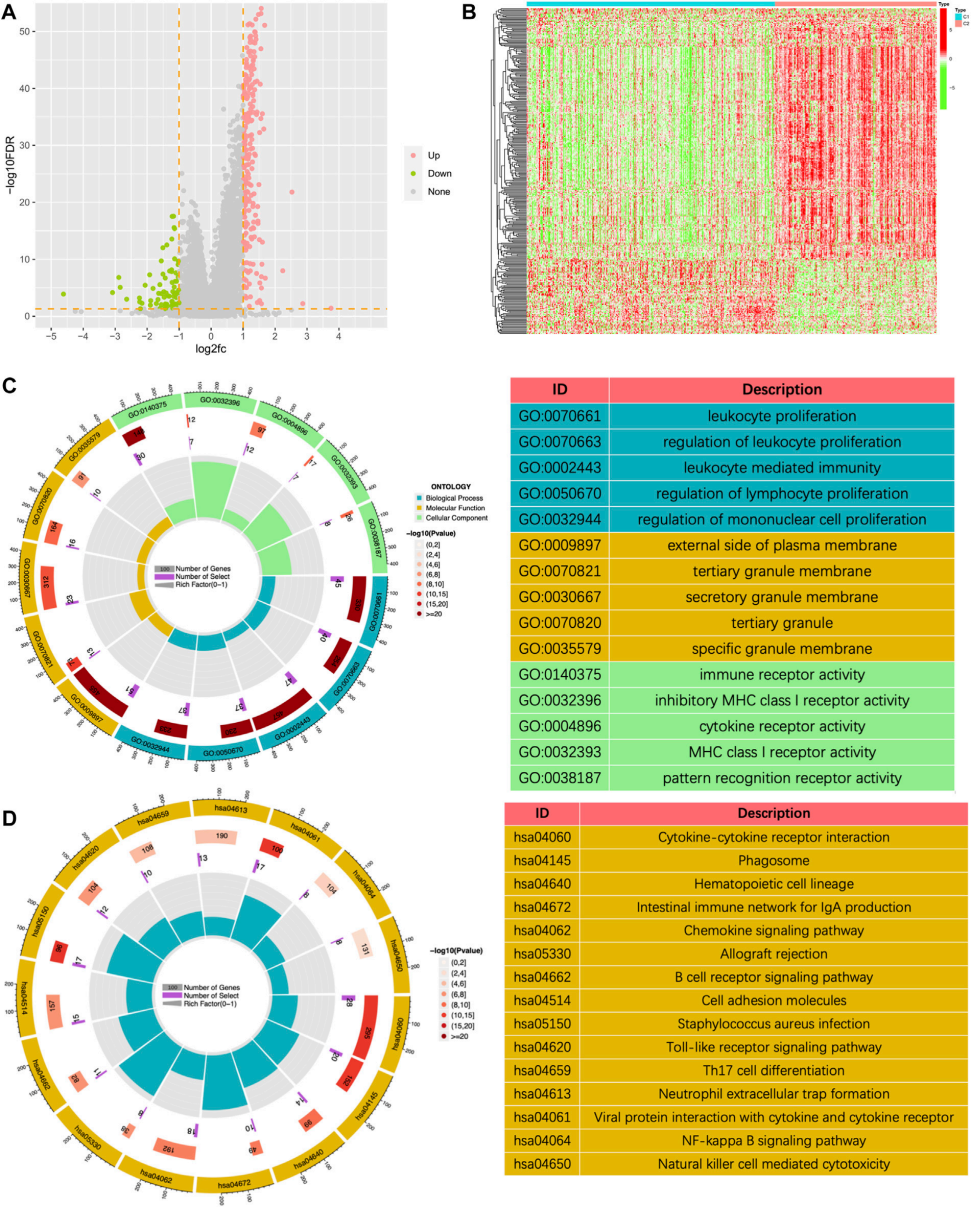
The volcano plot **(A)** and the heatmap **(B)** showing the differentially expressed genes (DEGs) in different clusters. **(C)** The GO analysis of the differentially expressed genes between different clusters. **(D)** The KEGG pathway enrichment analysis of differentially expressed genes between different clusters.

To further explore potential mechanisms, we conducted a differential analysis of the gene expression profiles between the two clusters and finally obtained 314 differentially expressed genes (DEGs). Figures 6A, B showed a volcano plot of the difference analysis and a heat map of DEGs between the two groups. GO and KEGG enrichment analyses were performed based on these 314 genes. The results of GO enrichment analysis mainly enriched in immune-related biological processes such as leukocyte proliferation, regulation of leukocyte proliferation, leukocyte mediated immunity, regulation of lymphocyte proliferation, and regulation of mononuclear cell proliferation (Figure 6C). The results of KEGG enrichment analysis also included cytokine receptor interaction, Phagosome, Intestinal immune network for IGA production, Chemokine signaling pathway, B cell receptor signaling pathway, etc. (Figure 6D). All the above results showed that tumor immunity was significantly different between the two subgroups classified by the NMF algorithm.

### Construction and validation of immune-related risk signature

**FIGURE 7:**
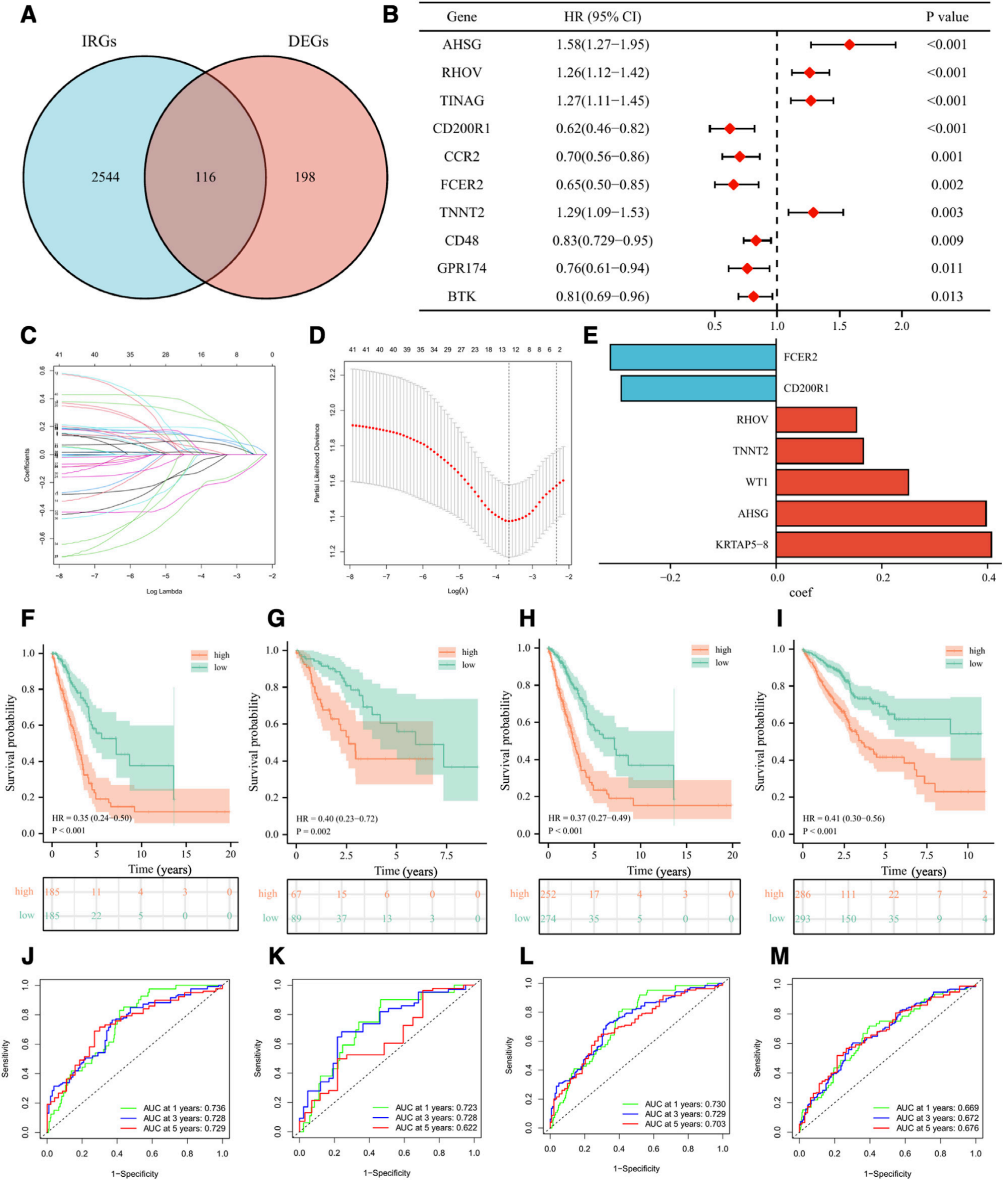
Development and validation of the immune-related signature. **(A)** Venn diagram displaying a total of 116 immune-related differentially expressed genes resulting from the intersection of 314 differentially expressed genes (DEGs) with 2660 immune-related genes (IRGs). **(B)** The forest plot displaying the HR (95% CI) and *p*-values for selected differentially expressed genes between different clusters using the univariate Cox regression analysis (top 10, according to *p*-value). **(C, D)** 12 candidate genes were obtained by LASSO regression analysis. **(E)** The coefficients of the 7 genes in the signature. The Kaplan–Meier curve survival analysis for LUAD patients stratified by the risk score in the TCGA training set **(F)**, the TCGA validation set **(G)**, the overall TCGA set **(H)**, the external validation set (GSE72094 and GSE41271) **(I)**. ROC analysis at 1, 3, and 5 years of LUAD patients in the TCGA training set **(J)**, the TCGA validation set **(K)**, the overall TCGA set **(L)**, and the external validation set (GSE72094 and GSE41271) **(M)**.

To better classify the above subgroups for clinical treatment guidance and quantify the specific risk score for each LUAD patient, we intersected 314 differentially expressed genes (DEGs) with 2660 immune-related genes (IRGs) and finally obtained 116 immunologically relevant differentially expressed genes (IR-DEGs) (Figure 7A). In the training set, we performed univariate Cox regression on the above IR-DEGs, and 44 genes with significant prognostic values were identified (Figure 7B). Then, we performed LASSO regression analysis to further screen the 44 IR-DEGs described above to refine the predictive signature (Figures 7C, D) and obtained 12 candidate genes. The list of the 116 IR-DEGs and the 44 genes with significant prognostic values were shown in Supplementary Table S7. To construct the final signature, a multivariate Cox regression analysis was performed on these 12 genes, with the risk score (RS) for each sample multiplying the expression of the final 7 genes by the coefficients of their multivariate cox regression (Figure 7E).

Based on the median value of RS in the training set, we divided LUAD patients in the training set, the validation set, and the external validation set (GSE72094 and GSE41271) into high-risk and low-risk groups. As was shown in the K-M survival analysis, the patients of the high-risk group had significantly worse OS than the low-risk group in either cohort (Figures 7F–I). For the training set, the AUC at 1-year, 3-year, and 5-year were 0.74, 0.73, and 0.73, respectively. For the validation set, the AUC at 1-year, 3-year, and 5-year were 0.72, 0.73, and 0.62. For the whole set, the AUC at 1-year, 3-year, and 5-year were 0.73, 0.73, and 0.70. As for the external validation set, the AUC at 1-year, 3-year, and 5-year were 0.67, 0.67, and 0.68 (Figures 7J–M). Besides, we validated our signature in different clinical subgroups (based on age, gender, smoking status, and tumor clinical stage), and found that patients in the low-risk group had a better prognosis than those in the high-risk group (Supplementary Figure S3). All the results confirmed that our risk signature had a good predictive ability.

### Correlation analysis of clinical characteristics and construction of nomogram

**FIGURE 8:**
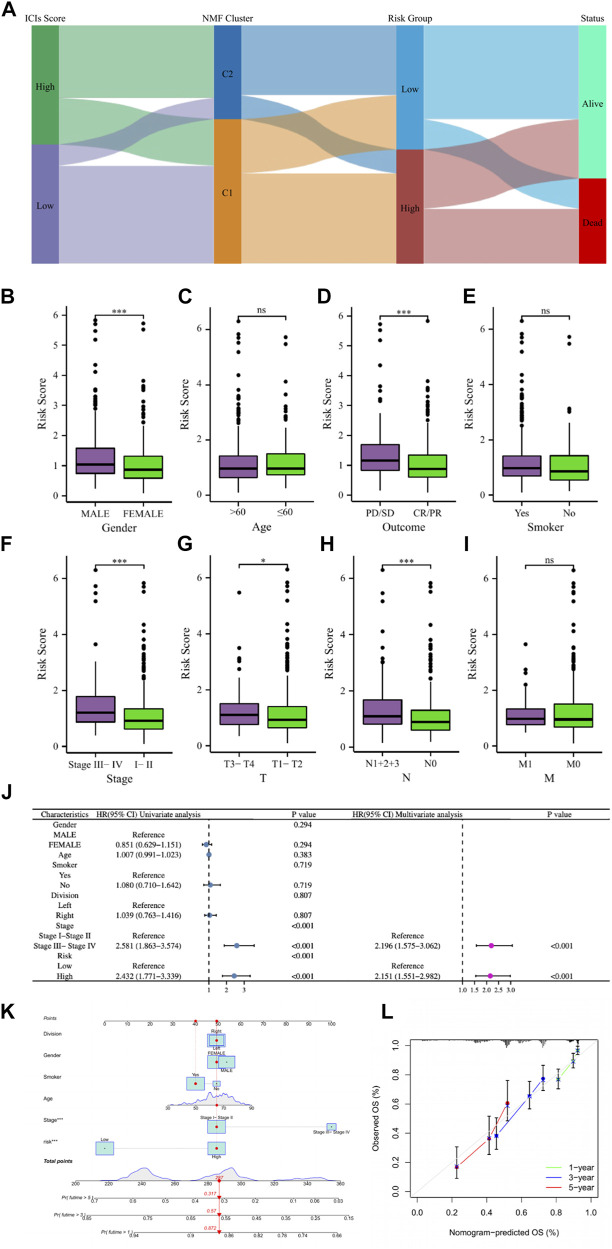
**(A)** The Sankey diagram revealed the potential connection between ICs score, NMF cluster, risk score, and survival status. **(B–I)** The comparisons of the risk score in LUAD patients with different gender, ages, main treatment outcomes, smoking status, clinical stage, T stage, N stage, and M stage. **(J)** Univariate and multivariate Cox regression analyses showed that risk score is an independent prognostic factor of LUAD. **(K)** The nomogram combining risk score and other clinicopathological parameters was developed to predict 1-, 3-, and 5-year survival. **(L)** Calibration curves showing the predictions of the nomogram that we established for 1-, 3-, and 5-year overall survival. ns, not significant; * p < 0.05 ; *** p < 0.001 .

LUAD patients in the above different classification modes can be intuitively reflected in the Sankey diagram (Figure 8A). Clinical correlation analysis results showed that male, PD/SD, late clinical stage, higher T stage, and higher N stage LUAD patients had higher risk scores, while clinical characteristics such as patient age, smoking, and M stage did not appear to be associated with risk score (Figures 8B–I). Supplementary Figure S4 showed comparations of the tumor environment and immune cell infiltration of the two risk groups. Univariate and multivariate cox analyses were utilized to further determine whether the risk score is an independent prognostic factor for LUAD, and the results showed that the risk score based on 7 gene expression was an independent prognostic factor for LUAD patients, and the clinical stage was also an independent prognostic factor (Figure 8J). The C-index and ROC curves showed that the predictive ability of the risk score was better than age, gender, smoking, lung cancer location, clinical stage, and other clinical characteristics (Supplementary Figure S5). To further provide a more accurate clinical prediction protocol, we constructed a prognostic nomogram based on the risk score and the patient’s clinical characteristics, which visually showed the 1-year, 3-year, and 5-year estimated survival rates of LUAD patients (Figure 8K). As demonstrated by the calibration curves, there was good agreement between the predicted patient survival and the actual survival (Figure 8L), which also indicates that our nomogram had a better prognostic value for LUAD patients.

### Enrichment analysis based on risk signature

**FIGURE 9:**
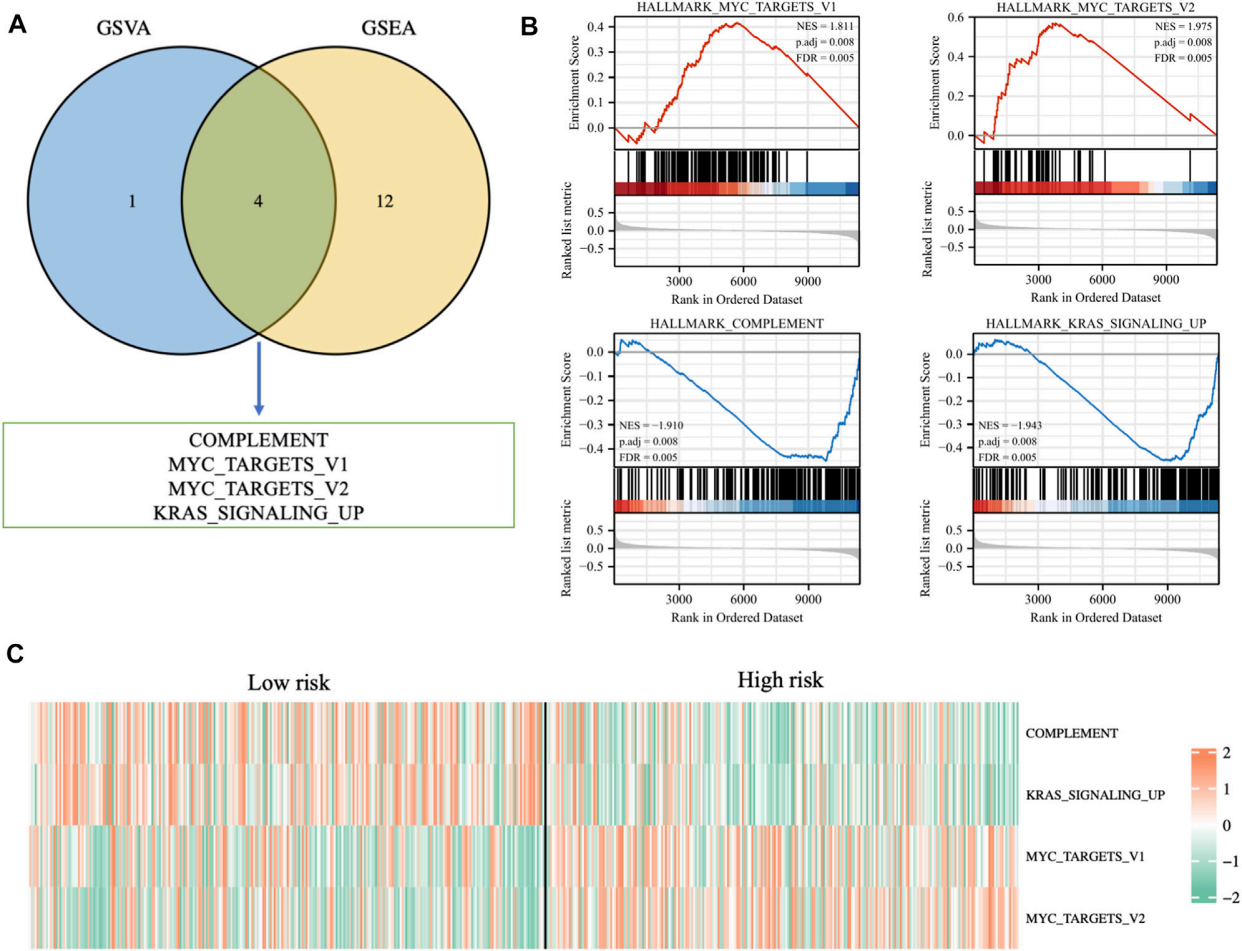
**(A)** Venn diagram displaying a total of four pathways resulting from the intersection of GSVA enrichment analysis with GSEA enrichment analysis based on differentially expressed genes in different risk groups. **(B)** GSEA enrichment analysis of the four pathways. **(C)** The heat map displaying GSVA enrichment scores of the four pathways of each patient.

In order to further explore the potential biological mechanisms that lead to so many differences between the two risk groups, GSEA enrichment analysis and GSVA enrichment analysis were performed based on the Hallmarks gene set (h.all.v7.2.symbols.gmt) in the MSigDB (https://www.gsea-msigdb.org/gsea/msigdb/index.jsp) database. The two types of enrichment analysis showed that compared with the tumors of patients in the low-risk group, there were more obvious features of MYC_TARGETS_V1, MYC_TARGETS_V2, and more deficient COMPLEMENT and KRAS_SIGNALING in the tumors of high-risk patients (Figures 9A, B). Figure 9C showed the heat map of the GSVA enrichment scores of the four characteristics of each patient.

### Guiding significance of risk signature for immunotherapy of LUAD patients

**FIGURE 10:**
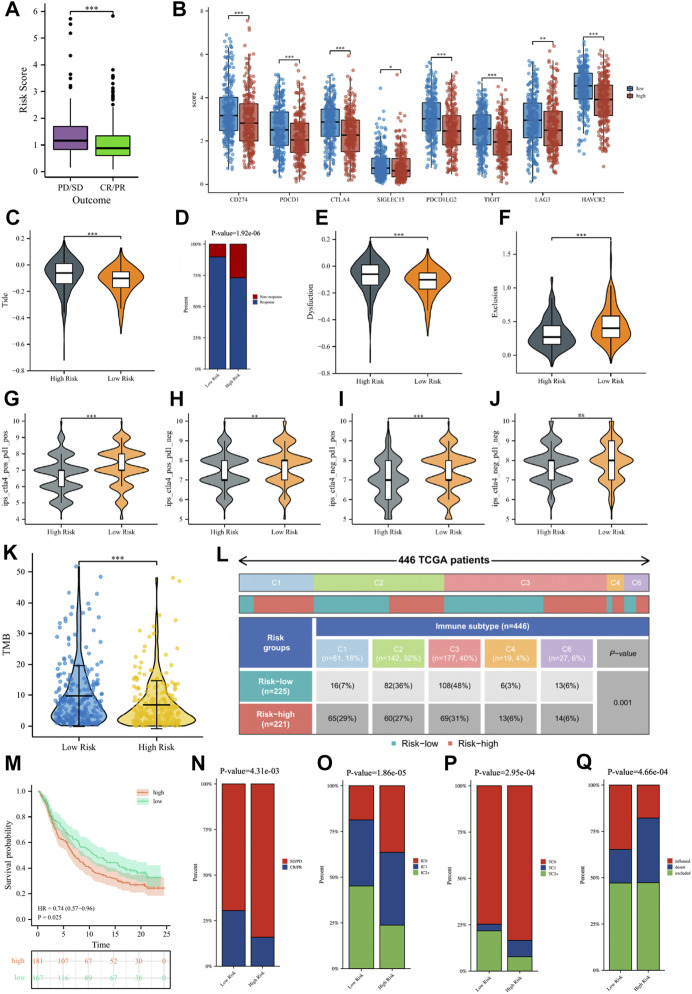
**(A)** The comparisons of the risk score in TCGA-LUAD patients with treatment outcomes. **(B)** The boxplot displaying the difference in immune checkpoint genes between different risk groups. The comparisons of the TIDE score **(C)**, Dysfunction score **(E)**, and Exclusion score **(F)** between different risk groups. **(D)** Percentage of immunotherapy response among risk groups of LUAD patients. The comparison of immunophenotype score (IPS) between different risk groups. **(G)** CTLA4+_PD1+, **(H)** CTLA4+_PD1−, **(I)** CTLA4−_PD1+, **(J)** CTLA4−_PD1−. **(K)** The comparisons of tumor mutational burden (TMB) of patients in different risk groups. **(L)** Comparison of the differences in immune subtype between different risk groups. **(M)** The Kaplan–Meier curve survival analysis between the high- and low-risk groups in the IMvigor210 cohort. Predictive value of risk score for immunotherapy response in the IMvigor210 cohort. **(N)** The percentage of immune response type among risk groups of patients in the IMvigor210 cohort. **(O)** The percentage of immune cell (IC) level type among risk groups of patients in the IMvigor210 cohort. **(P)** The percentage of tumor cell (TC) level type among risk groups of patients in the IMvigor210 cohort. **(Q)** The percentage of immune subtypes among risk groups of patients in the IMvigor210 cohort. Specimens were scored as immunohistochemistry IC0, IC1, IC2, or IC3 if <1%, ≥1% but <5%, ≥5% but <10%, or ≥10% of IC were PD-L1 positive, respectively. Specimens were scored as immunohistochemistry TC0, TC1, TC2, or TC3 if <1%, ≥1% but <5%, ≥5% but <50%, or ≥50% of TC were PD-L1 positive, respectively. ns, not significant; * p < 0.05 ; ** p < 0.01 ; *** p < 0.001 .

In the TCGA cohort, 439 LUAD patients received one or more treatment regimens, including 331 CR/ PR patients and 108 PD/SD patients. Comparing the risk scores of the two groups of patients, we noticed that PD/SD patients had significantly higher risk scores than CR/PR patients (Figure 10A). To better provide individualized treatment guidance for each LUAD patient, the role of the risk score in immunotherapy was further comprehensively analyzed. Figure 10B showed that a variety of classical immune checkpoint molecules, including CD274, CTLA4, PDCD1, and SIGLEC15, were more highly expressed in low-risk patients, which seems to suggest that the application of corresponding immune checkpoint inhibitors to such patients was more beneficial. According to the TIDE algorithm, low-risk patients had significantly lower Tide scores and Dysfunction scores and significantly higher Exclusion scores than high-risk patients, indicating that the possibility of immune escape in such patients is lower, and the efficacy may be better when using immunotherapy (Figures 10C, E, F). Correspondingly, for patients receiving immunotherapy, we can see that the percentage of patients responding to treatment in low-risk patients was significantly higher than that in high-risk patients (Figure 10D). Figures 10G–J showed a comparison of IPS scores between the two groups, where low-risk patients had significantly higher three IPS scores than high-risk patients, further suggesting that low-risk patients may be more sensitive to immunotherapy. TMB is a potential indicator for evaluating immunotherapy, and it has been reported that the higher the TMB of a tumor, the more neoantigens it is exposed to, and therefore more easily recognized and eliminated by the immune system. We found that the tumors of patients in the low-risk group had higher TMB (Figure 10K), so low-risk patients were more likely to benefit from immunotherapy, which also verified the above conclusion. Figure 10L showed the ratio of TCGA immune subtypes in patients with different risk groups, and significant differences in the immunophenotype did exist among patients in different risk groups.

Subsequently, we verified the response of immunotherapy in the IMvigor210 cohort. Figure 10M showed that the high-risk patients in the IMvigor210 cohort had worse OS than low-risk patients, consistent with the TCGA cohort and the GEO cohort. For immunotherapy, the percentage of low-risk patients with a good outcome of immunotherapy was significantly higher than that of high-risk patients (Figure 10N). The percentages of immune cells and tumor cells expressing PD-L1 were higher in low-risk patients than in high-risk patients (Figures 10O, P). Inflammatory-immune subtype analysis showed that low-risk patients had a higher percentage of “immune-inflamed” tumors and lower percentages of “immune-desert” and “immune-excluded” tumors compared to high-risk patients (Figure 10Q). The ROC curves showed that the predictive ability of the risk score in predicting the response of IO treatment was better when compared with PD-L1 levels (Supplementary Figure S6).

### Guiding significance of risk signature for chemotherapeutic and targeted therapy of LUAD patients

**FIGURE 11:**
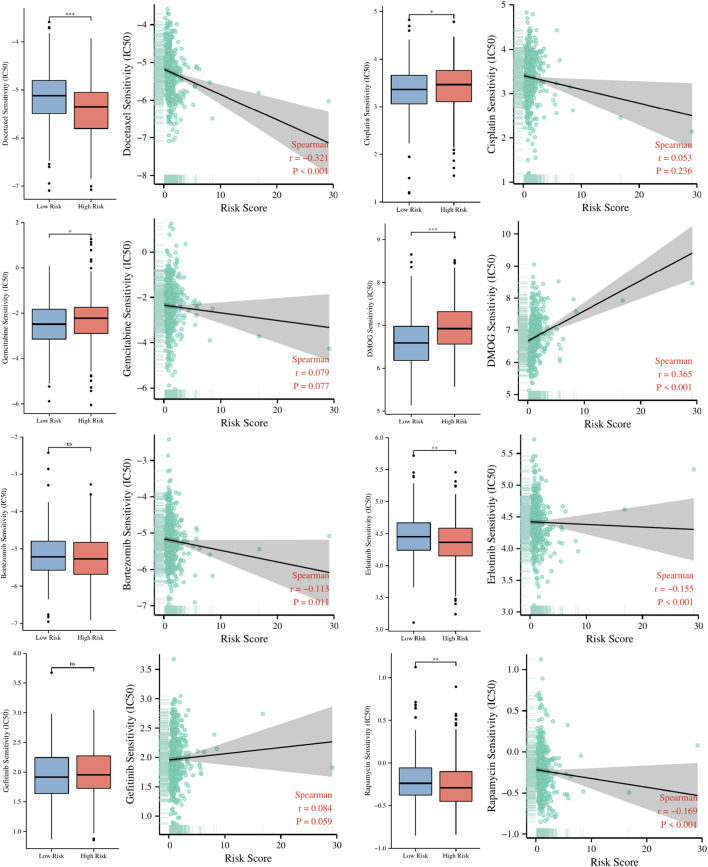
The relationship between risk scores and IC50 values of several common chemotherapy drugs. ns, not significant; * p < 0.05 ; ** p < 0.01 ; *** p < 0.001 .

To explore the guiding role of the risk signature in chemotherapeutic and targeted therapy for LUAD patients, we evaluated the relationship between risk scores and IC50 values of several common chemotherapy drugs such as cisplatin, docetaxel, gemcitabine, DMOG, rapamycin, Bortezomib, Erlotinib, Gefitinib. The results in Figure 11 indicate that patients in the high-risk group may be more sensitive to docetaxel, rapamycin, and Erlotinib, while patients in the low-risk group may benefit more from cisplatin, gemcitabine and DMOG.

### The RHOV gene is closely related to the development of LUAD

**FIGURE 12:**
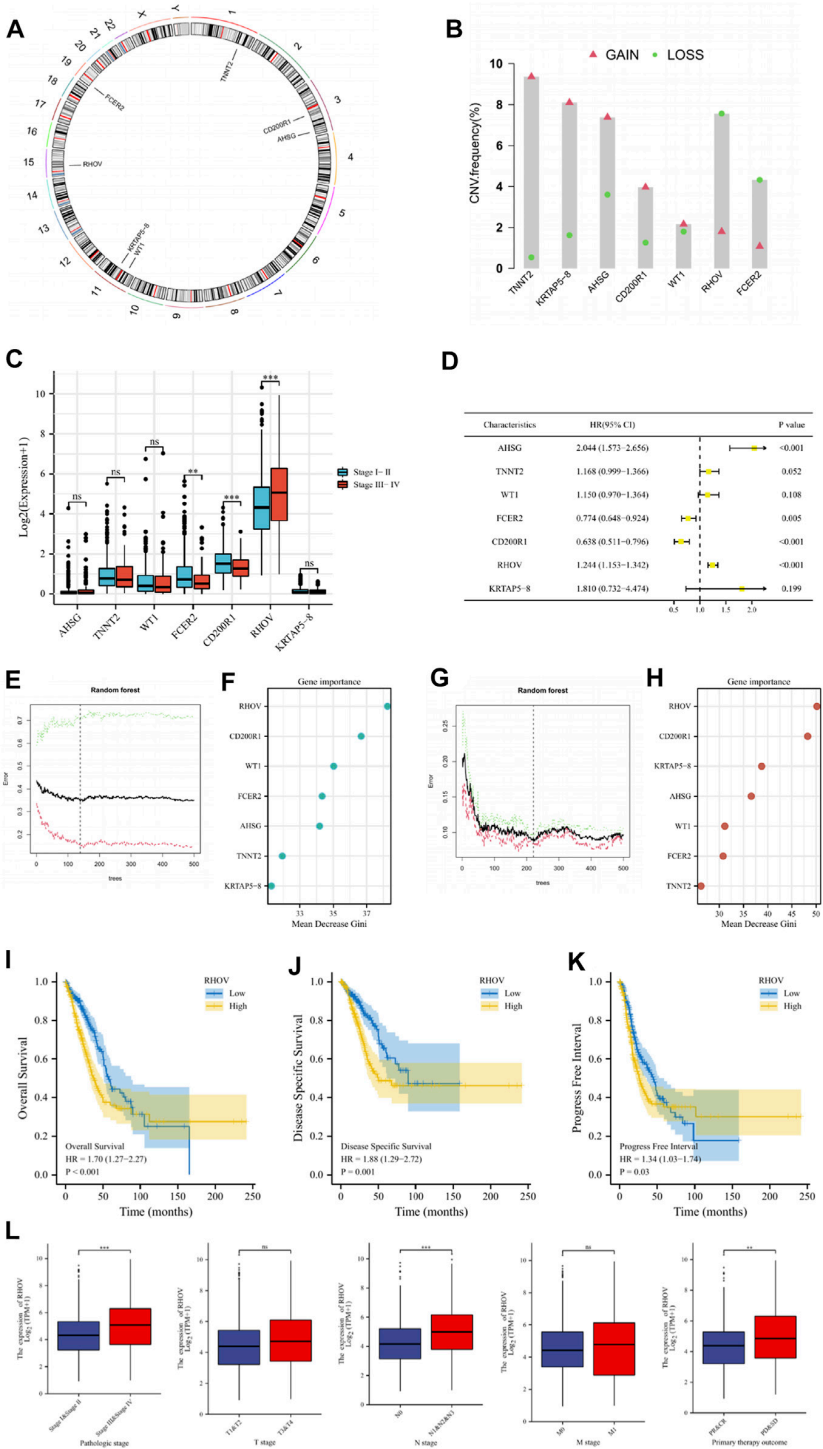
**(A)** Circus plots of chromosome distributions of selected genes from the risk model. **(B)** Frequencies of gain and loss for selected genes from the risk model. **(C)** The comparisons of risk model gene expression in patients with different clinical stages. **(D)** Univariate Cox regression analysis was performed on these risk model genes. **(E–H)** To further evaluate the importance of these genes for the prognostic contribution of LUAD patients, we performed a random forest analysis of these genes based on risk scores and patient survival status and found that the mean decrease Gini of RHOV was higher in both analyses. **(I–K)** The Kaplan–Meier curve survival analysis showing the relationship between the expression of RHOV and overall survival (OS), disease-specific survival (DSS), and progress-free interval (PFI). **(L)** The expression of RHOV in TCGA-LUAD patients with different pathologic stages, T stage, N stage, M stage, and primary therapy outcomes. ns, not significant; ** p < 0.01 ; *** p < 0.001 .

The chromosomal locations of the 7 genes in the risk signature were respectively shown in Figure 12A. Analysis of copy number variation frequencies showed that all seven signature genes exhibited significant CNV alterations (Figure 12B). In addition, FCER2, CD200R1, and RHOV in the signature were strongly associated with the clinical stage of the tumor (Figure 12C), and univariate cox analysis showed that except for TNNT2, WT1 and KRTAP5-8, the remaining signature genes were closely correlated with the prognosis of LUAD patients (Figure 12D). To further evaluate the importance of these genes for the prognostic contribution of LUAD patients, we performed a random forest analysis of these genes based on risk scores and patient survival status and found that the mean decrease Gini of RHOV was higher in both analyses (Figures 12E–H). In view of the important impact of RHOV on the survival and prognosis of LUAD patients, we conducted an in-depth study on it. Through K-M survival analysis, we found that LUAD patients with high RHOV expression had significantly worse OS, DSS, and PFI than LUAD patients with low RHOV expression (Figures 12I–K). The results of clinical correlation analysis showed that LUAD patients with late clinical stage, late N stage, and poor treatment effect had higher RHOV expression levels, which further confirmed that RHOV expression was closely related to the occurrence and development of LUAD (Figure 12L).

### Experimental verification

**FIGURE 13:**
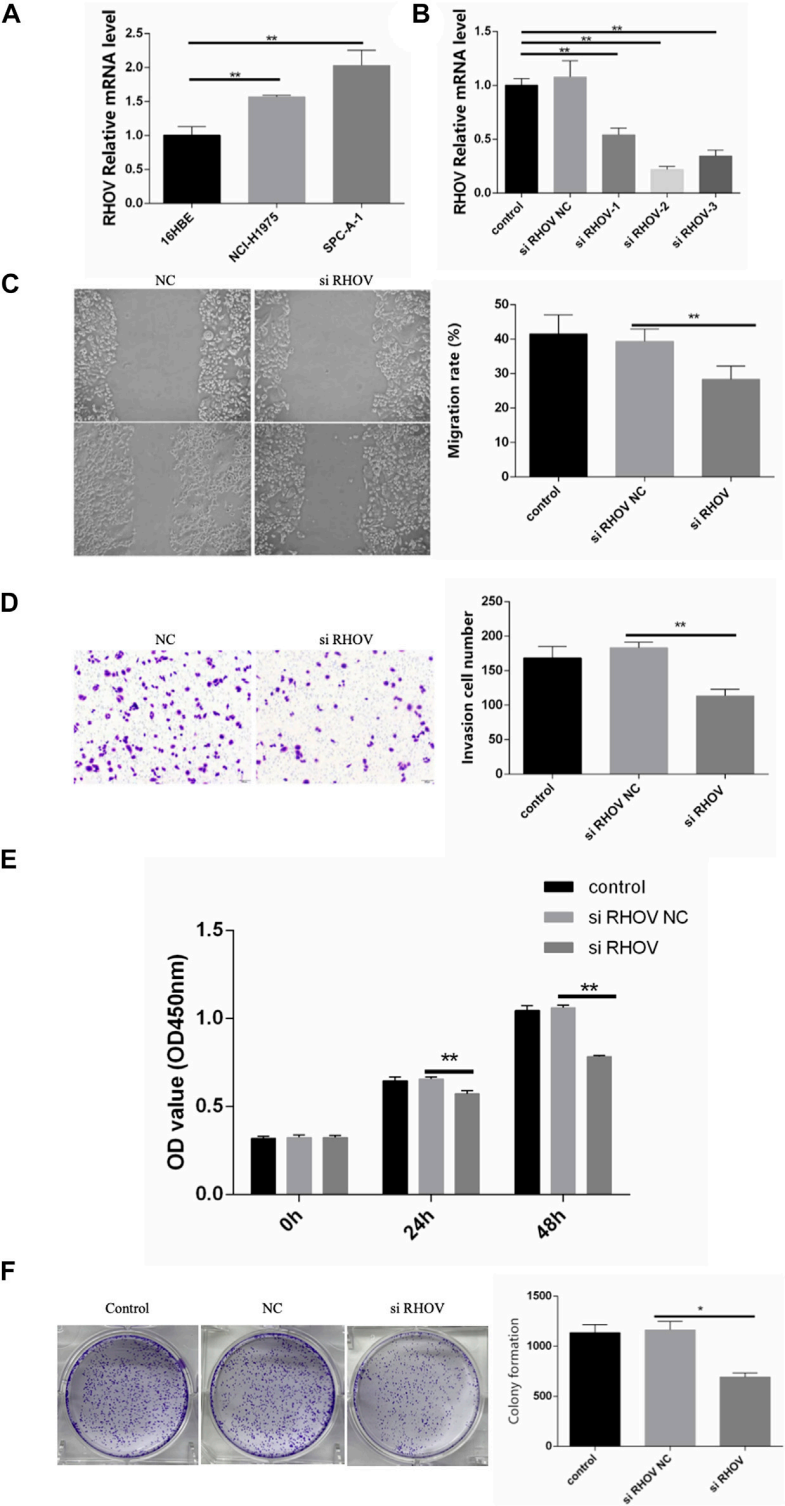
**(A)** The histogram showed the relative expression levels of RHOV evaluated by RT-qPCR in the 16HBE cells, SPC-A-1 cells, and NCI-H1975 cells. **(B)** Using RT-qPCR to evaluate the efficiency of gene knockdown by siRNA in the SPC-A-1 cells. **(C, D)** The effect of RHOV on cell migration was studied by scratch assay and transwell assay. **(E)** CCK-8 assay results showed the relative proliferation the siRNA-RHOV cells and the siRNA-NC cells. **(F)** The results of Cell colony formation assay. ns, not significant; * p < 0.05 ; ** p < 0.01 ; *** p < 0.001 .

We performed RT-qPCR to validate the expression of RHOV in 16HBE cells, SPC-A-1 cells, and NCI-H1975 cells. As shown in Figure 13A, compared with 16HBE cells, the expression levels of RHOV were significantly increased in SPC-A-1 cells and NCI-H1975 ( p<0.05 ), which was consistent with our previous analysis results. Besides, we also selected RHOV in the SPC-A-1 cells to further verify the accuracy of our model. The SPC-A-1 cells were transfected with siRNA-RHOV and siRNA- NC respectively. The results of RT-qPCR showed that the expression level of RHOV in the siRNA-RHOV group was significantly reduced when compared with the blank control group and the siRNA-NC group (Figure 13B). Subsequently, we performed a scratch assay and a transwell assay to evaluate the effect of the knockdown of RHOV on the migration of the SPC-A-1 cells. After knocking out RHOV, a decrease in migration ability was observed (Figures 13C, D). We also performed a proliferation assay. As shown in Figure 13E, after transfection of SPC-A-1 cell lines with siRNA-RHOV, the cell growth was significantly reduced at 24 and 48 h. Finally, the rate of cell colony formation was detected respectively and the knockdown of RHOV reduced the energy metabolism of SPC-A-1 cells and affected the effect of colony formation (Figure 13F).

## Discussion

The incidence of lung cancer has increased worldwide in recent years. Lung adenocarcinoma is the most common subtype of lung cancer and the 5-year survival rate for lung adenocarcinoma is very frustrating. Surgical lobectomy is the main treatment for LUAD at present, but the results are unsatisfactory due to the limited surgical outcomes and malignant nature of LUAD. Since patients with refractory malignancies including lung cancer benefit greatly from immune checkpoint inhibitors (Brahmer et al, 2015; Hellmann et al, 2017), immunotherapy is emerging as a new treatment option for cancer patients. As the key to immunotherapy, immune checkpoint molecules can play a critical role in the immune environment homeostasis by regulating the activation or inhibition of immune cells (Yu et al, 2020). So far, several ICIs targeting immune checkpoints, including Pembrolizumab, have shown tremendous potential in the treatment of various malignant tumors such as LUAD, and have been approved by the FDA for clinical treatment (Zhai et al, 2020). Unfortunately, although ICIs showed better efficacy and fewer side effects in LUAD patients, most patients did not benefit from ICIs (Schoenfeld and Hellmann, 2020). Some scholars have proposed that we may not be able to accurately identify patients who may be clinically eligible for immunotherapy only by the expression levels of classic ICGs such as CTLA4, PD-1, and PD-L and comprehensive analysis of ICGs in tumor tissues may be able to assess the responsiveness of patients more accurately to immunotherapy (Topalian et al, 2016).

In this study, we collected as many ICGs as possible as mentioned in the published literature. LUAD patients in TCGA and GEO datasets were scored according to a total of 82 ICGs expression levels, and module genes closely related to ICs scores were obtained by the WCGNA algorithm. Based on these genes, we classified LUAD patients into two subgroups with significant differences in prognosis and immune characteristics by NMF algorithm. Survival analysis showed that OS and DSS in the C2 group were significantly better than those in the C1 group. Tumor microenvironment and immune cell infiltration analysis showed that the immune and matrix scores were lower in C1 tumors, and the infiltration abundance of immune cells such as CD4 + T, CD8 + T, and B cells was also lower. Moreover, for the vast majority of ICGs differentially expressed in the two types of tumor tissues, their expression levels were significantly lower in C1 tumor tissues than in C2. The results of IPS immunotherapy analysis also confirmed that the response of C2 patients to ICIs did seem to be better than that of C1 patients. Enrichment analysis showed that the differentially expressed genes between the two subtypes were enriched in a variety of immune-related biological processes and signaling pathways.

A hierarchical signature consisting of 7 genes was constructed by Cox and LASSO algorithms based on the differentially expressed immune-related genes between the above two subgroups. LUAD patients were divided by the signature into two groups: low-risk patients and high-risk patients. Survival analysis showed that patients in the low-risk group had better OS than those in the higher-risk group in the TCGA and GEO patient cohorts. Clinical correlation analysis showed that the risk score was closely related to the gender, clinical stage, T stage, and N stage of LUAD patients. Independent prognostic analysis has shown that risk score and clinical stage were independent risk factors for LUAD patients. Integrating the above risk factors, the nomogram was constructed to predict the prognosis of LUAD patients, and the results were in good agreement with the actual survival rate of patients. To further guide the precise treatment of LUAD patients, we evaluated their responsiveness to immunotherapy through the TIDE algorithm, IPS algorithm, and TMB analysis and the results showed that low-risk patients seemed to be more likely to benefit from immunotherapy. According to the study of Thorsson et al (2018) on tumor immunotyping in TCGA, we found that the proportion of patients with C1 and C4 tumors in low-risk patients was lower than that in high-risk patients, and the proportion of patients with C2 and C3 tumors in low-risk patients was higher. As for the IMvigor210 cohort, we further verified the above analysis results. The response of patients in the high-risk group to immunotherapy was worse than that of patients in the low-risk group. As was shown in the immune subtypes analysis, “immune inflamed” tumors were more common in low-risk patients, while “immune desert” tumors were less common in high-risk patients.

As for the GSEA and GSVA enrichment analysis, the tumors of high-risk patients had obvious characteristics of the Myc pathway, and the tumors of low-risk patients had obvious characteristics of the complement system and KRAS signaling pathway. The Myc family of transcription factors consists of c-Myc, N-Myc, and L-Myc, and high expression of Myc contributes to tumorigenesis, including cell growth and metabolism of tumor cells, reduction of cell adhesion and metastasis, unrestricted proliferation, and inhibition of differentiation (Scognamiglio et al, 2016). As an important part of innate immunity, the complement system can effectively remove foreign bodies and maintain homeostasis. It has been reported that the complement system is not only involved in the killing and monitoring of tumor cells, but also in the process of promoting tumorigenesis (Li et al, 2018; Qian et al, 2019). Complement regulatory proteins such as CD35, CD46, CD55, and CD97 can inhibit the cytolysis of complement and evade immune surveillance. KRAS is a murine sarcomatoid virus oncogene and is responsible for controlling the path of regulating cell growth. When KRAS is mutated, it can lead to abnormal protein function and disorder of intracellular signal transduction, which leads to the continuous proliferation of tumors. The study of Sunaga et al (2011) showed that the detection rate of KRAS carcinogenic mutations in LUAD patients was > 25%, and these mutations predicted poor patient outcomes.

For the seven genes in the signature, we found FCER2, CD200R1, and RHOV closely correlated to the prognosis and clinical stage of LUAD patients. RHOV was identified as the most critical gene in this signature. Peng et al (2011) found that RHOV was differentially expressed in prostate cancer, and its expression level was closely related to the occurrence and development of tumors. In an earlier study, RHOV was considered to be overexpressed in NSCLC and may be a potential prognostic or diagnostic indicator for NSCLC (Shepelev and Korobko, 2013). In recent years, the understanding of the relationship between RHOV and LUAD has been further deepened. Zhang et al (2021) found that RHOV is closely related to LUAD metastasis. By activating the Jun N-terminal Kinase (JNK)/c-Jun signaling pathway and regulating the expression of markers of epithelial-to-mesenchymal transition, RHOV overexpression promotes the proliferation, migration, and invasion of LUAD cells, which indicates a shorter survival time. Chen et al (2021) found that RHOV overexpression promoted lung cancer progression and EGFR-TKI resistance and this might result from the activation of the AKT/ERK pathway. In this study, we found that RHOV was highly expressed in tumors of LUAD patients, and RHOV overexpression indicates a worse survival outcome. RHOV is also closely correlated with the clinical features of LUAD patients. To further study the relationship between RHOV and the occurrence and development of lung cancer, we carried out cytological experiments. The results showed that RHOV is significantly overexpressed in human lung cancer cell lines and after inhibiting RHOV expression, the proliferation and migration capacity of tumor cells decreased. Until now, the mechanism of how RHOV plays a role in the occurrence and progression of LUAD is not very clear, which needs further exploration in the future.

Compared with some recent studies about lung cancer immunotherapy (Xu et al, 2020; Ling et al, 2021; Chen et al, 2022), our research adopts different grouping methods, and the analysis of immune microenvironment is more comprehensive, and our risk signature has a higher AUC. Besides, we also validated our risk model gene through experiments in cell lines.

In summary, we performed a comprehensive analysis of the expression profile of ICGs in LUAD tumor tissues and thus constructed an immune-related signature based on the global landscape of ICGs. Stratified by this signature, the high-risk and low-risk patients are different in prognosis prediction, clinical characteristics, and treatment sensitivity. According to the results of our model analysis, early identification, timely intervention, and individualized treatment of the two types of patients are helpful to improve their quality of life and long-term survival rate. Finally, our study provides an important theoretical basis for further study of the role of ICGs in LUAD.

## Data Availability

The datasets presented in this study can be found in online repositories. The names of the repository/repositories and accession number(s) can be found in the article/Supplementary Material.

## Supplementary material

SUPPLEMENTARY TABLE S1 The reference for the signature genes of 23 immune cells derived from the TISIDB database.

SUPPLEMENTARY TABLE S3 The 82 immune checkpoints-related genes (ICGs).

SUPPLEMENTARY TABLE S5 The genes belonging to each module of TCGA cohort, GSE72094 cohort and GSE41271 cohort and the results of the intersection of the three modules.

SUPPLEMENTARY TABLE S7 The list of the 116 IR-DEGs and the 44 genes with significant prognostic values (with the pvalues and the HR values).

SUPPLEMENTARY FIGURE S1 The box plot showing the differential expression of ICGs between different ICs score subgroups.

SUPPLEMENTARY FIGURE S2 The waterfall graphs showing the relationship between ICs score and the status of common driver gene mutation.

SUPPLEMENTARY FIGURE S3 The Kaplan–Meier survival analysis showing different survival outcomes for patients grouped by different clinical characteristics in the TCGA-LUAD dataset. **(A)** Age subgroup; **(B)** Gender subgroup; **(C)** Smoking status subgroup; **(D)** Clinical stage subgroup.

SUPPLEMENTARY FIGURE S4
**(A)** The comparisons of the stromal score, immune score, and ESTIMATE score between different risk groups. **(B)** The comparisons of tumor purity between different risk groups. **(C)** The pod plot displaying the difference in immune cell infiltration between different risk groups.

SUPPLEMENTARY FIGURE S5 The C-index **(A)** and ROC curves **(B)** showing the comparisons of the predictive ability of the risk score and age, gender, smoking, lung cancer location, and clinical stage.

SUPPLEMENTARY FIGURE S6 The ROC curves showing the comparison of the predictive ability of the risk score in predicting the response of IO treatment compared with PD-L1 levels.
