# Supplementary figures and images for: Identification of lung adenocarcinoma subtypes and predictive signature for prognosis, immune features, and immunotherapy based on immune checkpoint genes
Source: Front Cell Dev Biol. 2023 May 10;11:1060086. doi: 10.3389/fcell.2023.1060086 (PMC10206047; doi:10.3389/fcell.2023.1060086)

**A**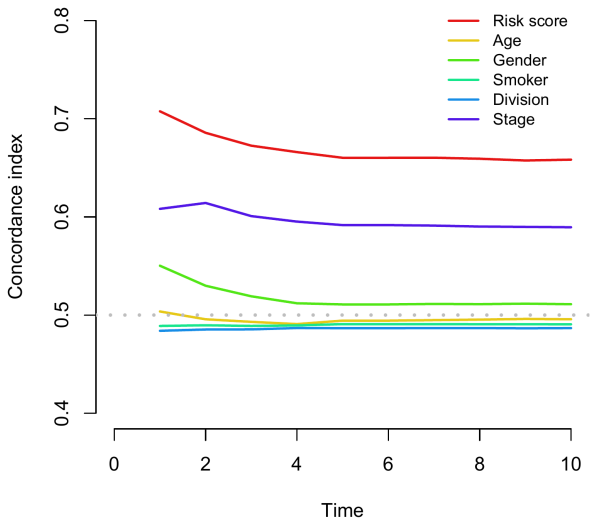**B**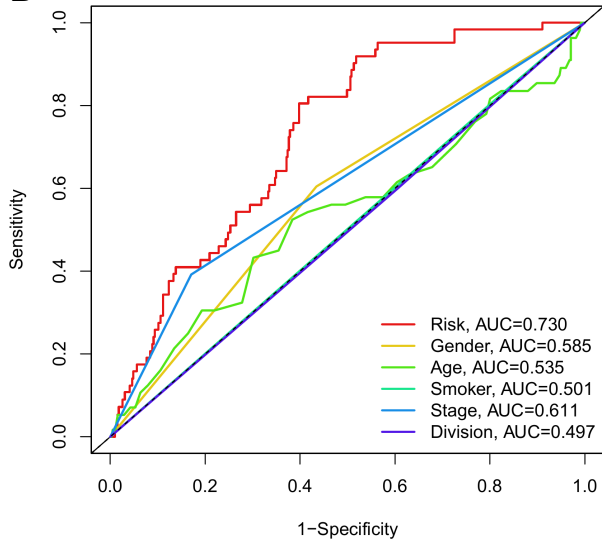

Supplement: Supplementary file 1 [file Image5.pdf]

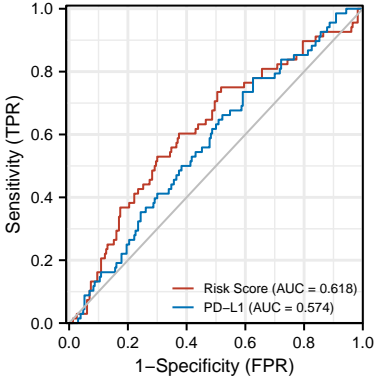

Supplement: Supplementary file 2 [file Image6.pdf]

**A**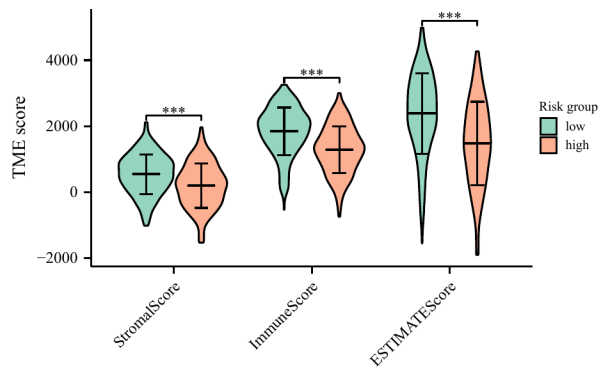**B**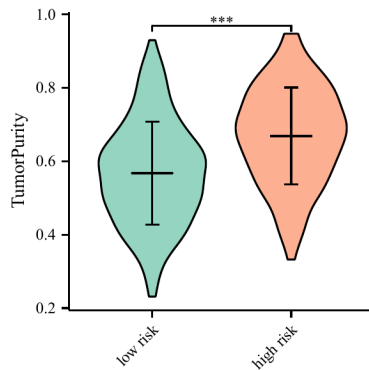**C**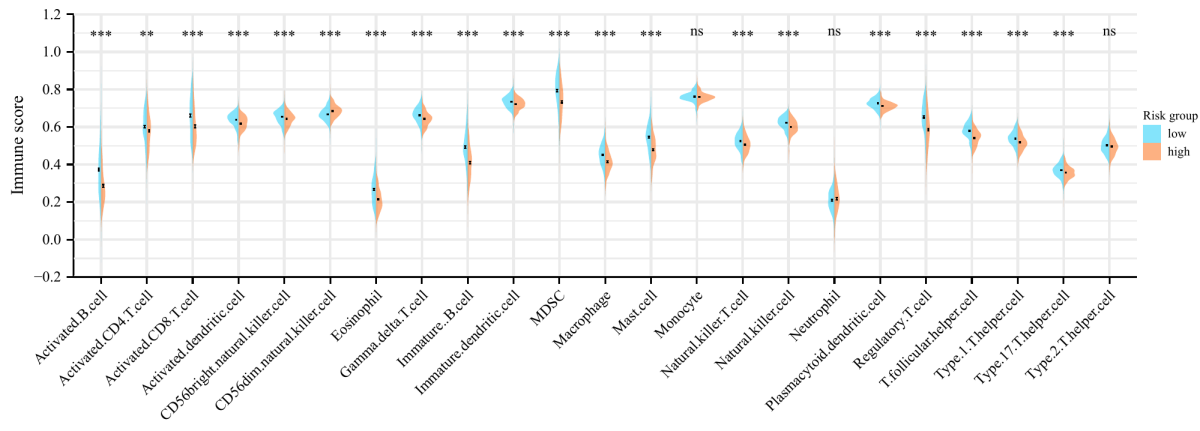

Supplement: Supplementary file 3 [file Image4.pdf]

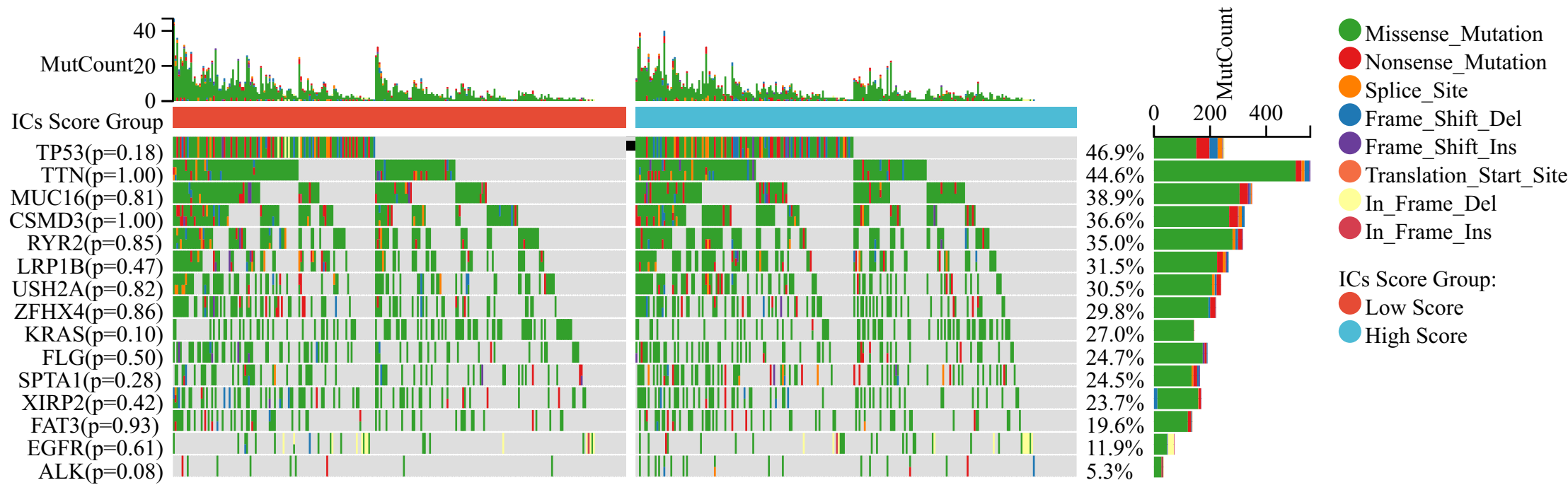

Supplement: Supplementary file 4 [file Image2.pdf]

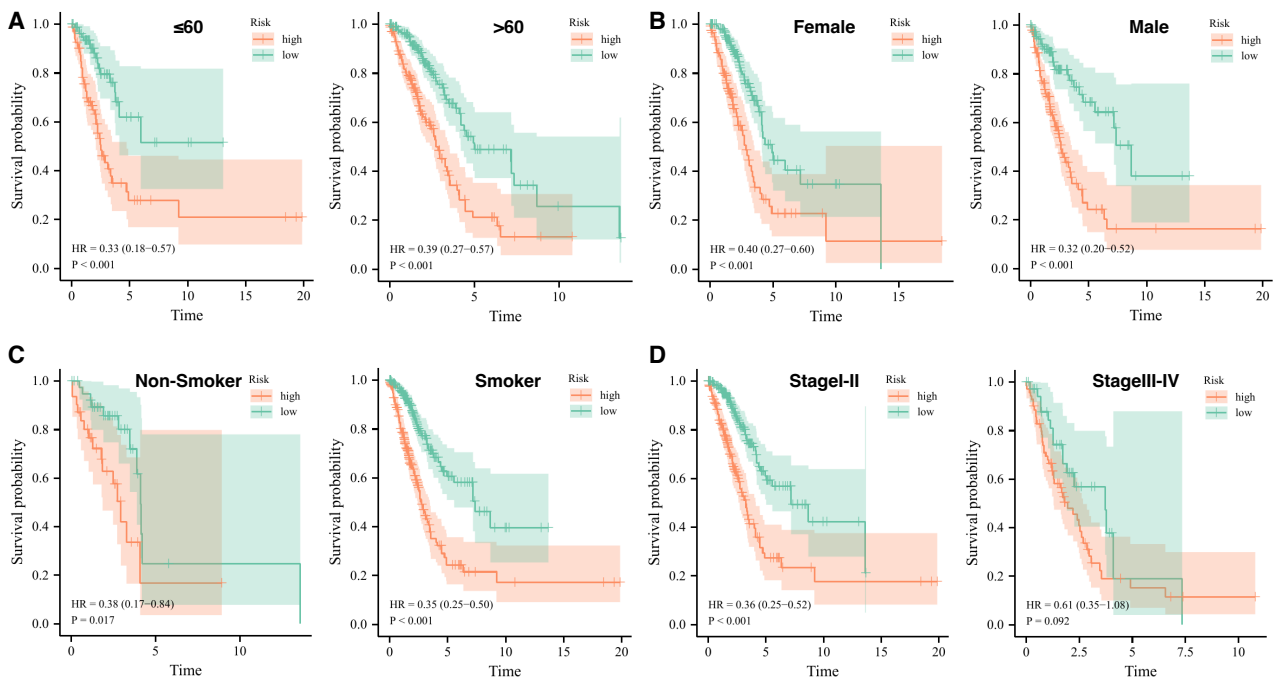

Supplement: Supplementary file 5 [file Image3.pdf]

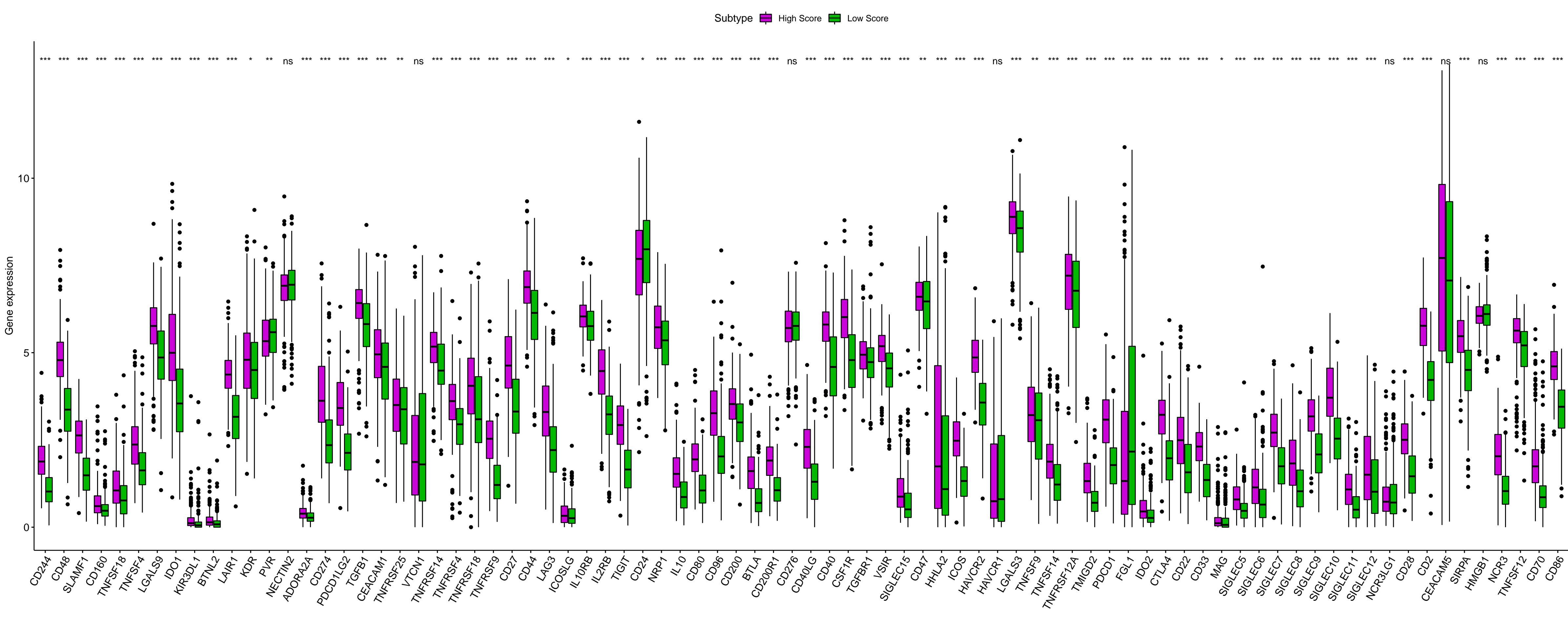

Supplement: Supplementary file 13 [file Image1.pdf]
