# Supplementary material for: Identification of lung adenocarcinoma subtypes and predictive signature for prognosis, immune features, and immunotherapy based on immune checkpoint genes
Source: Front Cell Dev Biol. 2023 May 10;11:1060086. doi: 10.3389/fcell.2023.1060086 (PMC10206047; doi:10.3389/fcell.2023.1060086)
Supplement: Supplementary file 6 [file Table2.DOCX]

| **Primer** | **Sequence (5’- 3’)** |
| --- | --- |
| h GAPDH_F | GCCTTCCGTGTCCCCACTGC |
| h GAPDH_R | GGCTGGTGGTCCAGGGGTCT |
| h RHOV_F | ACTGCGCTGGACACCTTC |
| h RHOV_R | ATGTTTTGAAAGGAGCTGGG |

**Primer sequences of related genes for RT-qPCR.**
