# Supplementary material for: Identification of lung adenocarcinoma subtypes and predictive signature for prognosis, immune features, and immunotherapy based on immune checkpoint genes
Source: Front Cell Dev Biol. 2023 May 10;11:1060086. doi: 10.3389/fcell.2023.1060086 (PMC10206047; doi:10.3389/fcell.2023.1060086)
Supplement: Supplementary file 7 [file Table3.DOCX]

| ICGs |
| --- |
| CD244 |
| CD48 |
| SLAMF1 |
| CD160 |
| TNFSF18 |
| TNFSF4 |
| LGALS9 |
| IDO1 |
| KIR3DL1 |
| BTNL2 |
| LAIR1 |
| KDR |
| PVR |
| NECTIN2 |
| ADORA2A |
| CD274 |
| PDCD1LG2 |
| TGFB1 |
| CEACAM1 |
| TNFRSF25 |
| VTCN1 |
| TNFRSF14 |
| TNFRSF4 |
| TNFRSF18 |
| TNFRSF9 |
| CD27 |
| CD44 |
| LAG3 |
| ICOSLG |
| IL10RB |
| IL2RB |
| TIGIT |
| CD24 |
| NRP1 |
| IL10 |
| CD80 |
| CD96 |
| CD200 |
| BTLA |
| CD200R1 |
| CD276 |
| CD40LG |
| CD40 |
| CSF1R |
| TGFBR1 |
| VSIR |
| SIGLEC15 |
| CD47 |
| HHLA2 |
| ICOS |
| HAVCR2 |
| HAVCR1 |
| LGALS3 |
| TNFSF9 |
| TNFSF14 |
| TNFRSF12A |
| TMIGD2 |
| PDCD1 |
| FGL1 |
| IDO2 |
| CTLA4 |
| CD22 |
| CD33 |
| MAG |
| SIGLEC5 |
| SIGLEC6 |
| SIGLEC7 |
| SIGLEC8 |
| SIGLEC9 |
| SIGLEC10 |
| SIGLEC11 |
| SIGLEC12 |
| NCR3LG1 |
| CD28 |
| CD2 |
| CEACAM5 |
| SIRPA |
| HMGB1 |
| NCR3 |
| [TNFSF12](file:///data/gene-symbol-report#!/hgnc_id/HGNC:11927) |
| CD70 |
| CD86 |
