# Supplementary material for: Identification of lung adenocarcinoma subtypes and predictive signature for prognosis, immune features, and immunotherapy based on immune checkpoint genes
Source: Front Cell Dev Biol. 2023 May 10;11:1060086. doi: 10.3389/fcell.2023.1060086 (PMC10206047; doi:10.3389/fcell.2023.1060086)
Supplement: Supplementary file 9 [file Table4.DOCX]

| Characteristics | TCGA（N=585） | GSE72094  （N=398） | GSE41271  （N=271） | IMVigor210  （N=348） |
| --- | --- | --- | --- | --- |
| Overall survival Status  Alive  Dead | 371  214 | 285  113 | 150  121 | 116  232 |
| Follow-up time  Median value  (Days) | 919.17 (0-7248) | 791.91 (3-2077) | 1319.00 (8-4027) | 3740.33 (72-8933) |
| Age  Median value  (Years) | 65.16 (33-88) | 69.30 (38-89) | 68.34 (31-94) | NA |
| Gender  Male  Female | 271  314 | 176  222 | 146  125 | 272  76 |
| Pathologic stage  Stage I  Stage II  Stage III  Stage IV  NA | 320  134  94  28  9 | 254  67  57  15  5 | 129  50  83  6  3 | 118  95  69  66  0 |

**Baseline characteristics of the LUAD patients in this study**
