# Supplementary material for: Identification of lung adenocarcinoma subtypes and predictive signature for prognosis, immune features, and immunotherapy based on immune checkpoint genes
Source: Front Cell Dev Biol. 2023 May 10;11:1060086. doi: 10.3389/fcell.2023.1060086 (PMC10206047; doi:10.3389/fcell.2023.1060086)
Supplement: Supplementary file 10 [file Table6.DOCX]

| Characteristics | Cluster 1  （N=319） | Cluster 2  （N=207） |
| --- | --- | --- |
| Overall survival Status  Alive  Dead | 130  189 | 149  58 |
| Follow-up time  Median value  (Days) | 902.38 (0-7248) | 879.23 (0-3674) |
| Age  Median value  (Years) | 65.07 (39-88) | 64.12 (33-85) |
| Gender  Male  Female | 162  157 | 84  123 |
| Pathologic stage  Stage I  Stage II  Stage III  Stage IV  NA | 94  30  27  6  0 | 117  46  29  10  5 |

**Baseline characteristics of the LUAD patients in the two clusters**
